# Deciphering the potential of the C-reactive protein-albumin-lymphocyte index as a prognostic biomarker in malignancy: a systematic review and meta-analysis

**DOI:** 10.3389/fonc.2026.1813296

**Published:** 2026-04-22

**Authors:** Ziqian Zhao, Hongyi Yuan, Haoyi Xu, Yuanjing Liu, Rui Xu, Kang Yan, Haojie Tang, Fanchao Dai, Yiming Wu, Chao Dong, Binlin Ma

**Affiliations:** The Clinical Medical Research Center of Breast and Thyroid Tumor in Xinjiang, Tumor Hospital Affiliated to Xinjiang Medical University, Urumqi, China

**Keywords:** biomarker, CALLY index, cancer, meta-analysis, prognosis

## Abstract

**Objective:**

The prognostic value of the pretreatment C-reactive protein–albumin–lymphocyte (CALLY) index for survival outcomes in patients with solid tumors is evaluated through a systematic review and meta-analysis.

**Methods:**

We conducted comprehensive searches in PubMed, EMBASE, Cochrane Library and Web of Science for all records published up to February 2026. Hazard ratios (HRs) with 95% confidence intervals (CIs) were retrieved. The associations between pretreatment CALLY and survival endpoints were synthesized using Stata 18.0 and Review Manager 5.4. Overall survival (OS) was the main endpoint; disease-free survival (DFS), progression-free survival (PFS), and recurrence-free survival (RFS) were also assessed.

**Results:**

Fifty-six reports (64 cohorts) comprising 26, 643 individuals were included. A higher CALLY index was correlated with superior OS (HR, 0.55; 95% CI, 0.50-0.60; P<0.00001), DFS (HR, 0.52; 95% CI, 0.46–0.58; P < 0.00001), PFS (HR, 0.39; 95% CI, 0.25-0.61; P<0.0001), and RFS (HR, 0.66; 95% CI, 0.60-0.72; P < 0.00001). Subgroup analyses revealed that effect estimates may be modified by sample size, follow-up duration, age, geographic region, CALLY cut-off values, cancer type, and tumor stage.

**Conclusions:**

A higher CALLY score is significantly associated with improved survival outcomes in patients with cancer, indicating its potential as a promising prognostic biomarker.

## 1 Introduction

Cancer constitutes one of the most pressing transnational health challenges, with approximately 20 million incident cases and 9.7 million deaths reported annually (1). Over the past decade, novel treatment modalities and anticancer agents have been developed; however, overall survival remains unsatisfactory. Therefore, identifying predictive factors for disease progression and survival may be valuable and may help clinicians develop the optimal treatment method for patients with cancer.

Inflammation and nutritional state play a pivotal role in tumor progression, treatment response, and survival (2, 3). Accordingly, several readily accessible blood-based indices have been investigated and associated with prognosis across multiple oncologic diseases, including the neutrophil-to-lymphocyte ratio (NLR), platelet-to-lymphocyte ratio (PLR), lymphocyte-to-C-reactive protein (CRP) ratio, lymphocyte-to-monocyte ratio (LMR), and the prognostic nutritional index (PNI) (4, 5).

The CALLY index is an integrated score that reflects nutritional reserve and systemic immune status. The CALLY index is generally calculated from serum albumin, peripheral blood lymphocyte count, and C-reactive protein (CRP) according to the following formula: CALLY = albumin × lymphocyte/CRP. Because the measurement units and conversion methods for albumin, lymphocyte count, and CRP may vary among studies, the absolute values and ranges of the CALLY index may also differ. A higher pretreatment CALLY index has been correlated with superior clinical outcomes across malignancies. In a single-center cohort of 253 patients receiving surgery for renal cell carcinoma (RCC), Hirata et al. reported that elevated pretreatment CALLY was linked with enhanced OS and prolonged PFS after a 3-year follow-up (6). Similarly, in a multicenter retrospective cohort of 192 patients with recurrent or metastatic head and neck squamous cell carcinoma (R/M HNSCC) who received nivolumab monotherapy between 2017 and 2023, Sato et al. noted that elevated pretreatment CALLY was linked with longer OS and PFS (7). Nevertheless, the clinical utility of CALLY across cancer types remains incompletely defined and warrants further investigation.

Two prior meta-analyses by Li et al. (8) and Zhang et al. (9) have synthesized 21 and 30 studies, respectively, and have reported that higher CALLY is correlated with improved OS and DFS/RFS in individuals with solid tumors. Since those analyses were published, additional clinical studies have accumulated, with findings that are not fully concordant. Notably, several common tumor types (e.g., urologic cancers and melanoma) have not been represented in earlier pooled analyses, and studies reporting progression-free survival have also been omitted. These issues may restrict the generalizability and robustness of the study results. Therefore, based on previous meta-analyses, this study aims to further include 26 newly published studies to comprehensively evaluate the prognostic utility of CALLY in individuals with cancer.

## 2 Materials and methods

### 2.1 Search strategy

The present study was designed and reported in accordance with the PRISMA 2020 guidelines, and the PRISMA 2020 checklist was provided in **Supplementary Table S1**. On February 1, 2026, we performed a comprehensive search of PubMed, Embase, Web of Science, and the Cochrane Library to locate studies meeting the eligibility criteria, with no language limits applied.For PubMed, the search terms included “CRP–albumin-lymphocyte index, “ “C-reactive protein-to-albumin-to-lymphocyte index, “ “CALLY, “ and related terminology. (**Supplementary Table S2**).

### 2.2 Study selection

Studies were identified using the PICOS framework. Inclusion required that studies (1) cancer was confirmed by histopathological or cytological examination; (2) participants were grouped according to pretreatment CALLY index levels; (3) at least one survival endpoint was reported, including OS, DFS, PFS, or RFS; and (4) HRs with 95% CIs and/or P values were provided. Studies were excluded if (1) hematologic malignancies were investigated; (2) the publication type was a conference abstract, review, case report, or letter; (3) HRs for OS/DFS/PFS/RFS were not reported; (4) the elements of the CALLY index were reported only as continuous variables. Additionally, when multiple reports originated from the same institution, we retained the one with the largest sample size. Study screening was independently performed by two authors, and the results were reconciled.

### 2.3 Data extraction

Data were independently retrieved by two investigators using a prespecified Excel form, and were consolidated by a third investigator. Any discrepancies were settled through consensus after discussion. The following information was collected: first author, publication year, country, study design, sample size, patient age, cancer type, study period, treatment modality, follow-up duration, TNM stage, CALLY cut-off value, timing of CALLY assessment, statistical model, and HRs with 95% CIs for OS, DFS, PFS, and RFS. Adjusted HRs with 95% CIs were preferentially extracted; unadjusted HRs were used only when adjusted estimates were unavailable, and the models were recorded for subgroup and meta-regression analyses.

### 2.4 Quality assessment

Study quality was appraised with the Newcastle–Ottawa Scale (NOS), which evaluates observational studies in three components: selection, comparability, and outcome. Studies scoring>6 points were assessed as methodologically rigorous. Two reviewers separately rated methodological rigor, and any discordance was reconciled through consensus after discussion (**Supplementary Table S3**).

### 2.5 Statistical analysis

HRs for OS and DFS, PFS, and RFS, together with 95% CIs, were obtained from each included study. Pooled effect estimates were obtained using a fixed- or random-effects model depending on heterogeneity across reports. All available HRs were included in the overall analysis, including multivariable-adjusted estimates and a small number of univariable HRs. A supplementary pooled analysis of HRs from multivariable Cox regression was also performed to assess the impact of unadjusted estimates. Heterogeneity was quantified with Cochran’s Q test and the Higgins I² statistic (10). Substantial heterogeneity was defined as I²>50% or P<0.05. A fixed-effect approach was chosen when heterogeneity was not notable; otherwise, a random-effects approach was adopted. Subgroup analyses and meta-regression were performed to investigate the causes of heterogeneity. Robustness was examined using leave-one-out sensitivity analyses. Publication bias was inspected by funnel plots and Egger’s test; when significant asymmetry was identified, trim-and-fill analyses were conducted to obtain bias-adjusted effects. All tests were two-sided, and P < 0.05 was defined as statistically significant. Statistical analyses were completed using STATA (version 18.0) and Review Manager (version 5.4).

## 3 Results

### 3.1 Study selection and study characteristics

**Figure 1:**
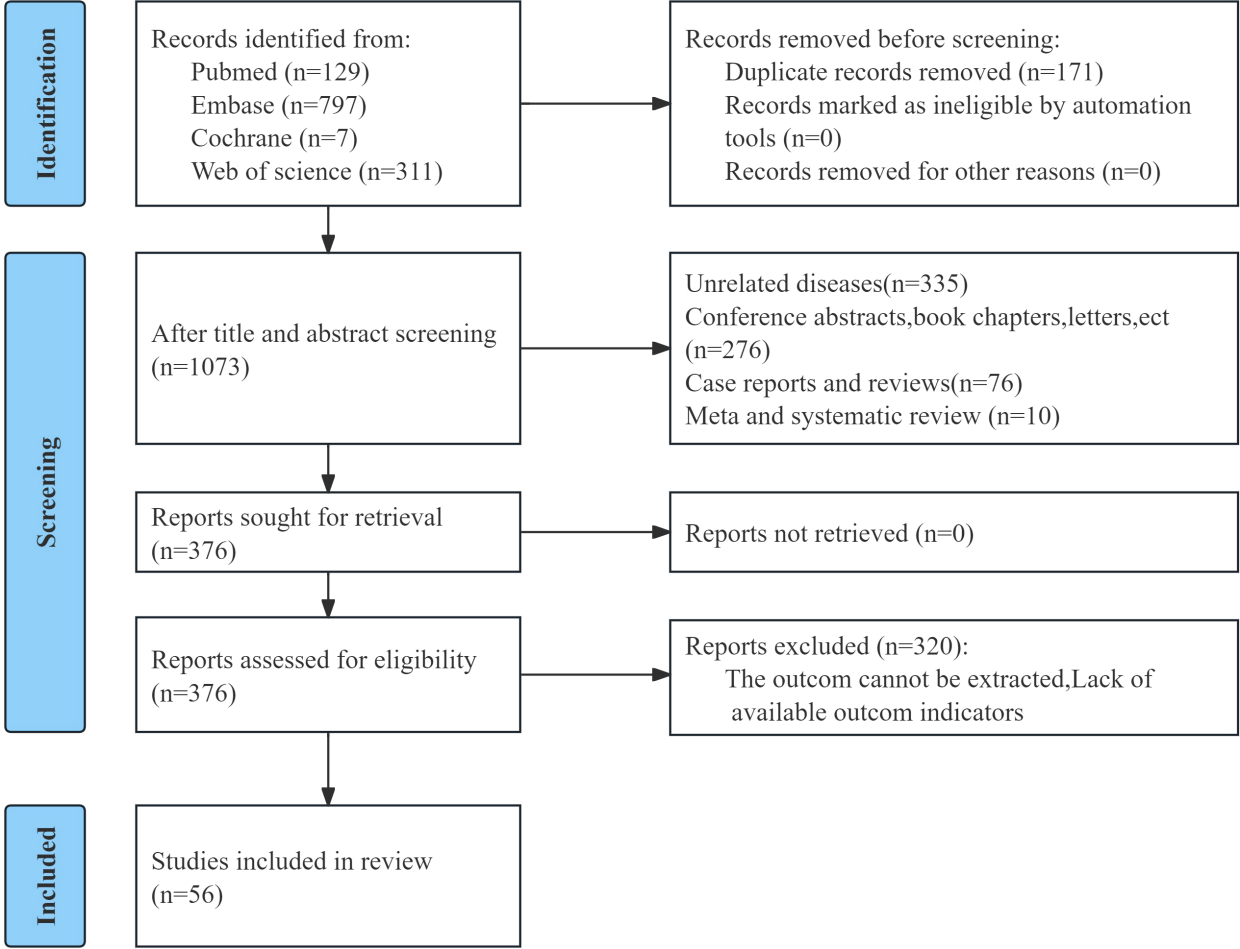
Flow chart of literature screening.

In total, 1, 244 records were identified, and 1, 171 records remained after duplicate removal. After title and abstract screening, 697 records were discarded. The full manuscripts of 376 records were reviewed, and 320 were excluded. Ultimately, 56 studies encompassing 26, 643 participants were integrated into the meta-analysis (**Figure 1**) (6, 7, 11–64). Across the 56 studies published between 2021 and 2026, 64 cohort groups were extracted. Most cohorts were derived from multicenter settings (n=22), followed by cohorts conducted in Japan (n=18). The included studies covered a diverse range of malignancies, including colorectal cancer (CRC; n=14), gastric cancer (GC; n=9), esophageal cancer (EC; n=6), lung cancer (n=6), liver cancer (n=7), HNSCC (n=5), urologic cancers (n=4), pancreatic ductal adenocarcinoma (n =4), cholangiocarcinoma (n=2), ovarian cancer (n=1), breast cancer (BC; n=1), melanoma (n=1), and retroperitoneal sarcoma (RPS; n=1). Sixty-three cohorts were retrospective, and one cohort was prospective. All cohorts were published in English, and study periods ranged from 1999 to 2025. Median age ranged from 50 to 77 years, and CALLY cut-off values ranged from 0.10 to 6.96. Among the 64 included comparisons, 55 reported multivariable-adjusted HRs, whereas 9 provided only univariable HRs (**Table 1**). The pooled analysis of DFS was based entirely on adjusted HRs. For OS, PFS, and RFS, both adjusted and unadjusted HRs were included in the overall analysis, and the supplementary analysis restricted to multivariable Cox regression yielded consistent results.

**Table 1:** Basic characteristics of the included literature.

Author	Study period	Region	Study design	Cancer type	Treatment method	Timing of detection	No. of patients	Gender (male/female)	Median follow-up (months)	Mean/median age	TNM stage	Cut-off	Outcomes
Liu, X.Y. 2023a (16)	2013-2018	Multicenter	Retrospective cohort	Non-small-cell lung cancer (NSCLC)	surgery and chemotherapy, radiation, or other anti-cancer treatments	Pre-treatment	1307	853/454	21.31	61.00	I-IV	1.32	OS
Liu, X.Y. 2023b (16)	2013-2018	Multicenter	Retrospective cohort	Non-small-cell lung cancer (NSCLC)	surgery and chemotherapy, radiation, or other anti-cancer treatments	Pre-treatment	557	NA	21.31	61.00	I-IV	1.32	OS
Nakashima, K. 2024 (26)	2011-2019	Japan	Retrospective cohort	Gastric cancer (GC)	surgery	Pre-treatment	175	119/56	NA	70.00	I-III	6.96	OS、DFS
Yao, Z.Y. 2025 (56)	2021-2024	China	Retrospective cohort	Advanced HER-2 negative gastric cancer	sintilimab combined with chemotherapy	Pre-treatment	200	125/75	14	60.00	III-IV	1.13	OS、PFS
Zhang, H.Y. 2023 (19)	2013-2018	Multicenter	Retrospective cohort	Gastric cancer (GC)	surgery and chemotherapy, radiation, or other anti-cancer treatments	Pre-treatment	684	479/205	NA	59.00	I-IV	1.12	OS
Iida, H. 2022a (12)	2011-2013	Multicenter	Retrospective cohort	Hepatocellular carcinoma (HCC)	surgery	Pre-treatment	267	NA	NA	69.00	I-IV	5.00	OS、RFS
Iida, H. 2022b (12)	2011-2013	Multicenter	Retrospective cohort	Hepatocellular carcinoma (HCC)	surgery	Pre-treatment	384	NA	NA	69.00	I-IV	5.00	OS、RFS
Hagiwara, K. 2024a (22)	2019-2022	Multicenter	Retrospective cohort	Recurrent or metastatic squamous cell carcinoma of the head and neck (R/M SCCHN)	Pembrolizumab-alone	Pre-treatment	119	95/24	8.60	72.00	NA	0.24	OS、PFS
Hagiwara, K. 2024b (22)	2019-2022	Multicenter	Retrospective cohort	Recurrent or metastatic squamous cell carcinoma of the head and neck (R/M SCCHN)	Pembrolizumab with chemotherapy	Pre-treatment	28	25/3	8.60	64.00	NA	0.34	OS、PFS
Çitakkul, I. 2025 (40)	2017-2025	Turkey	Retrospective cohort	Pancreatic ductal adenocarcinoma (PDAC)	surgery and adjuvant therapy	Pre-treatment	109	54/55	NA	64.00	I-III	1.03	OS
Hirata, H. 2025a (6)	2005-2023	Japan	Retrospective cohort	Renal cell carcinoma (RCC)	surgery	Pre-treatment	253	172/81	37.90	67.00	T1-T2	2.16	OS
Hirata, H. 2025b (6)	2005-2023	Japan	Retrospective cohort	Renal cell carcinoma (RCC)	surgery	Post-treatment	253	172/81	37.90	67.00	T1-T2	2.16	OS、PFS
Wang, S.Y. 2025 (7)	2010-2025	Multicenter	Retrospective cohort	Cancer patients with sarcopenia	surgery and chemotherapy, radiation, or other anti-cancer treatments	Pre-treatment	1389	723/666	NA	70.50	I-IV	2.25	OS
Sato, F. 2025 (7)	2017-2023	Multicenter	Retrospective cohort	Recurrent or metastatic squamous cell carcinoma of the head and neck (R/M SCCHN)	nivolumab	Pre-treatment	103	85/18	NA	68.00	NA	0.33	OS、PFS
Cetinayak, H.O. 2025 (38)	2010-2024	Turkey	Retrospective cohort	Hypopharyngeal squamous cell carcinoma	chemoradiotherapy	Pre-treatment	71	47/24	15.00	62.00	II-IV	1.47	OS、PFS
Lang, S.Q. 2025 (45)	2010-2021	China	Retrospective cohort	Intrahepatic cholangiocarcinoma (ICC)	surgery	Pre-treatment	200	122/78	NA	60.00	I-III	1.81	OS、RFS
Tsai, Y.T. 2022 (13)	2008-2017	China	Retrospective cohort	Oral cavity squamous cell carcinoma (OSCC)	surgery	Pre-treatment	279	249/30	48.10	56.00	I-IV	0.65	OS、DFS
Zhuang, J.R. 2024 (31)	2017-2018	China	Retrospective cohort	Breast cancer(BC)	surgery	Pre-treatment	174	0/174	NA	50.00	I-III	2.29	OS、DFS
Bahardoust, M. 2025 (35)	2012-2022	Multicenter	Retrospective cohort	Colorectal cancer (CRC)	surgery and adjuvant chemotherapy	Pre-treatment	1140	661/479	NA	57.60	I-IV	2.00	OS
Akgüner, G. 2025 (34)	2014-2024	Multicenter	Retrospective cohort	Metastatic renal cell carcinoma (mRCC)	surgery and chemotherapy, radiation, or other anti-cancer treatments	Pre-treatment	95	67/28	NA	62.15	I-IV	0.12	OS、PFS
Acar, C. 2025 (32)	2015-2024	Turkey	Retrospective cohort	Metastatic melanoma	anti-PD-1 monotherapy	Pre-treatment	92	53/39	NA	62.00	NA	2.00	OS、PFS
Takeda, Y. 2024 (29)	2010-2017	Japan	Retrospective cohort	Colorectal cancer (CRC)	surgery	Pre-treatment	589	348/230	63.80	69.00	II-III	2.00	OS、DFS
Fukushima, N. 2024 (21)	2010-2017	Multicenter	Retrospective cohort	Gastric cancer (GC)	surgery	Pre-treatment	826	594/232	5.80	68.00	I-III	2.00	OS、RFS
Xi, P. 2025 (27)	2009-2023	China	Retrospective cohort	Ampullary carcinoma (AC)	surgery	Pre-treatment	201	127/74	46.30	59.12	I-III	0.70	OS
Sun, J.K. 2025 (53)	2014-2023	China	Retrospective cohort	Colorectal cancer (CRC)	surgery	Pre-treatment	303	175/128	22.20	62.00	NA	NA	PFS
Kobayashi, Y. 2025 (44)	2014-2021	Japan	Retrospective cohort	Colorectal cancer (CRC)	surgery	Pre-treatment	178	92/86	42.30	77.00	I-III	1.20	OS、RFS
Mizota, K. 2026a (64)	2013-2018	Multicenter	Retrospective cohort	Non-small-cell lung cancer (NSCLC)	surgery	Pre-treatment	581	340/241	NA	70.00	I-II	6.20	OS、RFS
Mizota, K. 2026b (64)	2013-2018	Multicenter	Retrospective cohort	Non-small-cell lung cancer (NSCLC)	surgery	Pre-treatment	634	347/287	NA	70.00	I-II	6.20	OS
Zhu, L.X. 2025	2019-2024	China	Retrospective cohort	Metastatic pancreatic cancer	gemcitabine	Pre-treatment	252	168/84	NA	66.69	NA	0.27	OS
Wang, W. 2022 (14)	2010-2021	China	Retrospective cohort	Epithelial Ovarian Cancer	surgery	Pre-treatment	190	0/190	NA	57.50	I-IV	3.00	OS、RFS
Meng, P.Z. 2025a (49)	2016-2024	China	Retrospective cohort	Esophageal cancer (EC)	surgery	Pre-treatment	553	456/97	30.00	64.80	I-IV	2.55	OS、DFS
Meng, P.Z. 2025b (49)	2010-2018	China	Retrospective cohort	Esophageal cancer (EC)	surgery	Pre-treatment	104	85/19	37.00	60.10	I-IV	2.55	OS、DFS
Li, J.Q. 2025 (46)	2012-2020	China	Retrospective cohort	Colorectal cancer (CRC)	surgery	Pre-treatment	255	144/111	NA	62.58	I-III	6.79	OS、RFS
Furukawa, S. 2025 (42)	2013-2019	Japan	Retrospective cohort	Colorectal cancer (CRC)	surgery	Pre-treatment	223	121/102	NA	71.50	II-III	3.41	OS、RFS
Zhu, D. 2024 (30)	1999-2018	Multicenter	Retrospective cohort	Cancer	surgery and chemotherapy, radiation, or other anti-cancer treatments	Pre-treatment	3511	1671/1840	103.00	66.10	NA	NA	OS
Mashiko, T. 2025 (48)	2005-2020	Japan	Retrospective cohort	Pancreatic ductal carcinoma	surgery	Pre-treatment	109	41/68	49.00	69.00	IA-IB	4.40	RFS
Miyake, M. 2026 (64)	2000-2025	Multicenter	Retrospective cohort	Retroperitoneal sarcoma (RPS)	surgery	Pre-treatment	102	72/46	32.00	65.00	I-IV	2.70	OS、RFS
Seber, E.S. 2025 (51)	2012-2024	Turkey	Retrospective cohort	Extensive-stage small cell lung cancer (ES-SCLC)	platinum etoposide chemotherapy regimen without immunotherapy	Pre-treatment	167	153/14	6.00	62.00	NA	4.55	OS
Dirim, M.G. 2025 (41)	2010-2020	Turkey	Retrospective cohort	Hepatocellular carcinoma (HCC)	surgery and chemotherapy, radiation, or other anti-cancer treatments	Pre-treatment	199	153/46	56.90	63.00	NA	0.41	OS
Toda, M. 2025 (54)	2013-2017	Japan	Retrospective cohort	Gastric cancer (GC)	surgery	Pre-treatment	358	227/131	NA	70.00	I-III	2.00	OS、RFS
Tsunematsu, M. 2023 (17)	2002-2019	Japan	Retrospective cohort	Distal cholangiocarcinoma	surgery	Pre-treatment	143	66/77	42.00	68.00	I-IV	3.50	OS、DFS
Furukawa, K. 2023 (15)	2000-2018	Japan	Retrospective cohort	Colorectal liver metastasis	surgery	Pre-treatment	183	127/56	NA	65.00	NA	4.00	OS、DFS
Muller, L. 2021 (11)	2008-2013	Germany	Prospective cohort	Hepatocellular carcinoma (HCC)	transarterial chemoembolization (TACE)	Pre-treatment	280	234/46	NA	69.50	NA	1.00	OS
Cheng, H. 2025 (39)	2017-2024	China	Retrospective cohort	Non-small-cell lung cancer (NSCLC)	surgery	Pre-treatment	302	207/95	NA	60.00	I-III	1.39	OS
Zhang, Y.C. 2025 (57)	2020-2024	China	Retrospective cohort	Locally advanced rectal cancer (LARC)	neoadjuvant chemoradiotherapy (NACRT)	Pre-treatment	131	95/36	26.00	62.00	I-IV	1.47	OS、PFS
Zhao, H. 2025 (58)	2013-2022	China	Retrospective cohort	Liver cancer	surgery and chemotherapy, radiation, or other anti-cancer treatments	Pre-treatment	388	289/99	NA	57.40	I-IV	1.31	OS
Aoyama, T. 2024 (20)	2005-2020	Japan	Retrospective cohort	Esophageal cancer (EC)	surgery	Pre-treatment	180	155/25	NA	69.00	I-III	5.00	OS、RFS
Bekki, T. 2025a (36)	2010-2018	Multicenter	Retrospective cohort	Colorectal cancer (CRC)	surgery	Pre-treatment	1659	865/794	NA	67.00	I-III	3.35	OS、RFS
Bekki, T. 2025b (36)	2010-2018	Japan	Retrospective cohort	Colorectal cancer (CRC)	surgery	Pre-treatment	608	371/237	NA	67.00	I-III	3.35	OS、RFS
Hirata, H. 2024 (24)	2005-2023	Japan	Retrospective cohort	Renal cell carcinoma (RCC)	surgery	Pre-treatment	80	55/25	93.50	65.50	T3	1.28	PFS
Shiraishi, T. 2025 (52)	2016-2023	Multicenter	Retrospective cohort	Obstructive colorectal cancer	surgery	Pre-treatment	263	149/114	NA	69.00	NA	0.37	OS、RFS
Kawahara, S. 2024 (25)	2013-2022	Japan	Retrospective cohort	Pancreatic cancer	surgery	Pre-treatment	461	243/218	NA	71.00	I-III	1.90	OS、RFS
Sakurai, K. 2024 (28)	2014-2020	Japan	Retrospective cohort	Gastric cancer (GC)	surgery	Pre-treatment	563	343/220	NA	70.00	I-IV	1.19	OS
Matsui, S. 2026 (62)	2007-2022	Japan	Retrospective cohort	Intrahepatic cholangiocarcinoma (ICC)	surgery	Pre-treatment	131	87/44	NA	69.50	I-IV	3.00	OS、DFS
Zhu, M.L. 2025 (31)	2010-2015	China	Retrospective cohort	Breast cancer(BC)	surgery	Pre-treatment	187	0/187	NA	52.00	III	0.10	OS、DFS
47, R.Y. 2025a (47)	2008-2018	Multicenter	Retrospective cohort	Esophageal cancer (EC)	surgery	Pre-treatment	146	123/23	NA	69.00	I-IV	2.40	OS
Ma, R.Y. 2025b (47)	2008-2018	Multicenter	Retrospective cohort	Esophageal cancer (EC)	surgery	Pre-treatment	146	123/23	NA	69.00	I-IV	4.56	DFS
Hashimoto, I. 2024 (23)	2013-2017	Japan	Retrospective cohort	Gastric cancer (GC)	surgery	Pre-treatment	459	300/139	NA	65.00	I-III	3.28	OS、RFS
Okugawa, Y. 2024 (27)	2000-2001	Japan	Retrospective cohort	Gastric cancer (GC)	surgery	Pre-treatment	426	304/122	38.20	67.50	I-IV	4.93	OS、DFS
61 (61)	2010-2020	China	Retrospective cohort	Colorectal cancer (CRC)	surgery	Pre-treatment	957	516/441	NA	69.00	I-III	4.66	OS、DFS
43 (43)	2013-2023	Multicenter	Retrospective cohort	Esophageal cancer (EC)	surgery and chemotherapy, radiation, or other anti-cancer treatments	Pre-treatment	518	462/56	NA	69.00	I-IV	3.58	OS
18 (18)	2012-2020	Multicenter	Retrospective cohort	Colorectal cancer (CRC)	surgery and chemotherapy, radiation, or other anti-cancer treatments	Pre-treatment	1260	767/493	NA	60.00	I-IV	1.35	OS
33an, O. 2025 (33)	2014-2020	Turkey	Retrospective cohort	Early-stage Gastric cancer	adjuvant or perioperative chemotherapy	Pre-treatment	74	46/28	33.50	60.00	II-III	1.34	OS、RFS
Bjelanovic, J. 2025 (37)	2019-2024	Serbia	Retrospective cohort	Locally advanced rectal cancer (LARC)	chemotherapy	Pre-treatment	30	19/11	62.50	66.00	III	2.31	OS

### 3.2 Study quality

All included publications exhibited NOS scores ≥ 6, indicating generally high methodological quality. **Supplementary Table S1** summarizes the NOS ratings.

### 3.3 Meta-analysis results

#### 3.3.1 CALLY and OS

**Figure 2:**
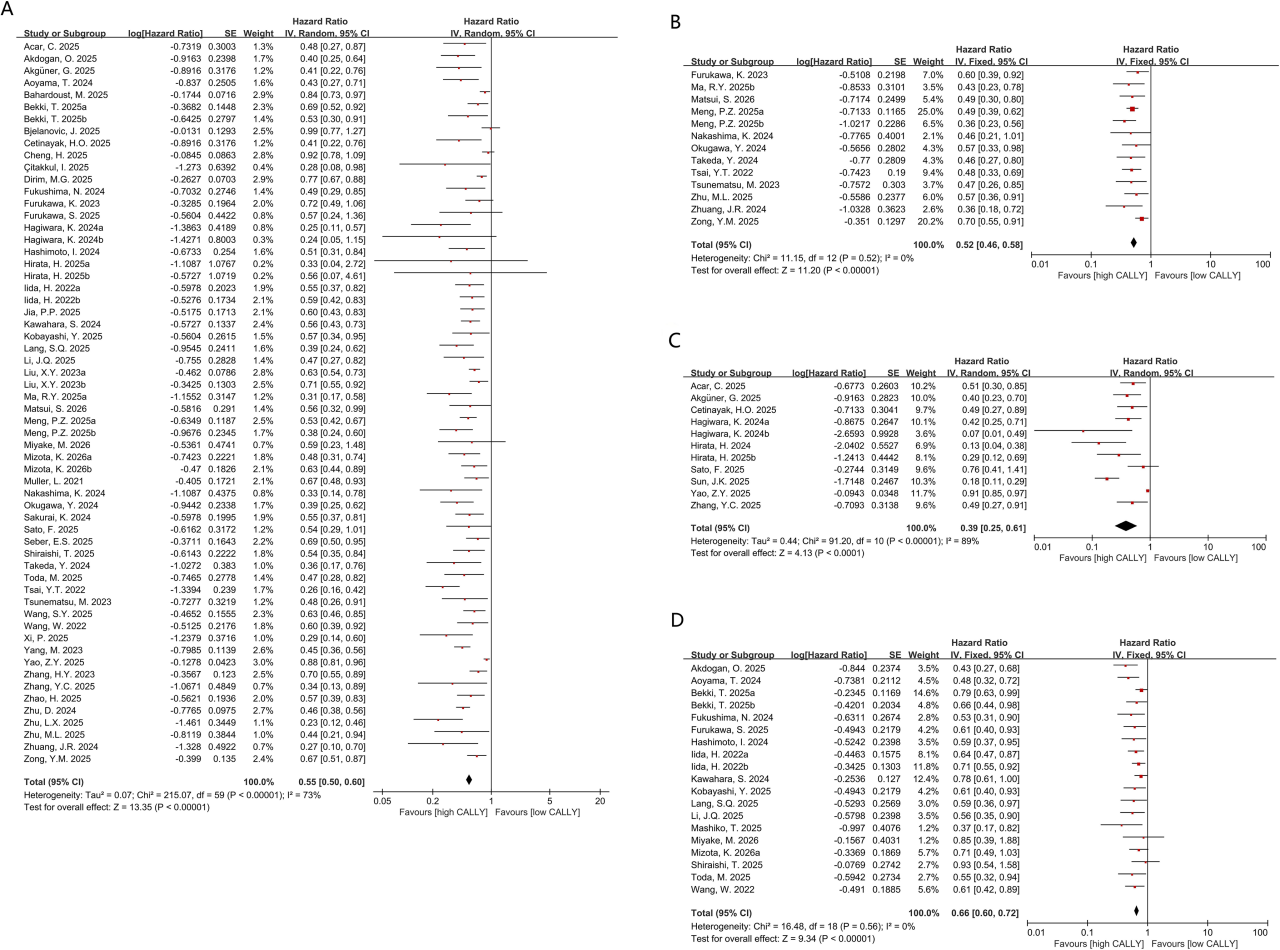
**(A)** Forest plots for the association between CALLY and OS; **(B)** forest plots for the association between CALLY and DFS; **(C)** forest plots for the association between CALLY and PFS;**(D)** forest plots for the association between CALLY and RFS.

OS was reported in 60 comparison groups. As shown in the forest plot (**Figure 2A**), higher pretreatment CALLY was linked to improved OS compared with lower CALLY (HR, 0.55; 95% CI, 0.50-0.60; P<0.00001). Considerable heterogeneity was noted (I²=73%, P<0.00001); therefore, a random-effects model was employed. To investigate explanatory factors of heterogeneity, subgroup analyses were undertaken. The correlation between higher CALLY and improved OS remained consistent across all prespecified subgroups (**Table 2**). Stratified analyses by age, region, cut-off value, tumor stage, and cancer type showed no significant heterogeneity in several subgroups (I² < 50%), suggesting that these factors might have contributed to between-study heterogeneity.

**Table 2:** Pooled HRs for OS in subgroup analyses.

Subgroup	OS			
	Study group	HR [95%CI]	*P* value	*I* ^2^
Total	60	0.55 [0.50, 0.60]	<0.00001	73%
Sample size				
<250	28	0.51 [0.44, 0.60]	<0.00001	73%
≥250	32	0.57 [0.51, 0.63]	<0.00001	67%
Follow-up				
<30 months	9	0.60 [0.48, 0.75]	<0.00001	78%
≥30 months	15	0.49 [0.39, 0.61]	<0.00001	78%
Mean/median Age				
<65	26	0.55 [0.48, 0.63]	<0.00001	81%
≥65	34	0.55 [0.50, 0.61]	<0.00001	37%
Region				
China	15	0.49 [0.39, 0.61]	<0.00001	86%
Japan	16	0.52 [0.46, 0.59]	<0.00001	0%
Other	8	0.64 [0.51, 0.79]	<0.0001	65%
Multicenter	21	0.57 [0.51, 0.64]	<0.00001	63%
CALLY cut-off				
<1	10	0.37 [0.24, 0.57]	<0.00001	82%
1-2	16	0.59 [0.51, 0.70]	<0.00001	81%
2-3	14	0.54 [0.43, 0.67]	<0.00001	71%
3-4	8	0.60 [0.51, 0.70]	<0.00001	0%
4	11	0.58 [0.51, 0.65]	<0.00001	9%
Treatment.				
Surgery	48	0.55 [0.50, 0.60]	<0.00001	63%
Other	12	0.53 [0.41, 0.69]	<0.00001	79%
Cancer type				
Gastric cancer	9	0.54 [0.41, 0.70]	<0.00001	81%
Colorectal cancer	13	0.62 [0.52, 0.74]	<0.00001	69%
Esophageal cancer	5	0.48 [0.40, 0.59]	<0.00001	22%
Hepatopancreatobiliary Cancer	12	0.53 [0.44, 0.64]	<0.00001	62%
Urologic cancer	3	0.41 [0.23, 0.73]	0.003	0%
Lung cancer	6	0.69 [0.57, 0.82]	<0.0001	67%
Head and Neck Squamous Cell Carcinoma	5	0.34 [0.25, 0.46]	<0.00001	9%
Other	7	0.50 [0.44, 0.58]	<0.00001	0%
Tumor stage				
Nonmetastatic	4	0.56 [0.42, 0.73]	<0.0001	0%
Metastatic	11	0.56 [0.43, 0.73]	<0.0001	77%
Mixed	45	0.54 [0.50, 0.60]	<0.00001	65%
Adjustment				
Univariate Analysis	8	0.72 [0.59, 0.87]	0.001	67%
Multivariate Analysis	52	0.52 [0.48, 0.58]	<0.00001	71%

Univariable meta-regression identified adjustment status and cancer type as the main sources of heterogeneity (**Table 3**), and these findings were confirmed by multivariable meta-regression, which showed both factors to be independent contributors (**Table 4**). When the analysis was restricted to studies reporting multivariable-adjusted HRs, the association remained statistically significant (HR = 0.52, 95% CI: 0.48-0.58, P < 0.00001) (**Supplementary Figure S1A**).

**Table 3:** Univariate meta-regression for the association of confounding factors and the hazard ratio for OS.

Variables	Coefficient	P value	95% CI
Sample size			
Per 1-patient increase	0.00003	0.629	[-0.00010, 0.00017]
Follow-up			
Per 1-month increase	-0.00085	0.812	[-0.00783, 0.00614]
Mean/Median Age			
Per 1-year increase	-0.002	0.819	[-0.019, 0.015]
CALLY cut-off			
Per 1-unit increase	0.005	0.857	[-0.047, 0.056]
Region			
Japan vs China	-0.021	0.869	[-0.272, 0.230]
Multicenter vs China	0.086	0.434	[-0.129, 0.301]
Other vs China	0.205	0.150	[-0.074, 0.484]
Tumor stage			
Non-metastatic vs Metastatic	-0.061	0.799	[-0.535, 0.412]
Mixed vs Metastatic	-0.055	0.654	[-0.293, 0.184]
Treatment method			
Other vs Surgery	0.051	0.651	[-0.169, 0.271]
Adjustment			
Multivariate vs Univariate	-0.292	0.010	[-0.514, -0.070]
Cancer type			
Colorectal cancer vs Gastric cancer	0.105	0.414	[-0.147, 0.358]
Esophageal cancer vs Gastric cancer	-0.192	0.245	[-0.515, 0.131]
Hepatopancreatobiliary Cancer vs Gastric cancer	-0.053	0.689	[-0.314, 0.208]
Urologic cancer vs Gastric cancer	-0.311	0.381	[-1.008, 0.385]
Lung cancer vs Gastric cancer	0.188	0.188	[-0.092, 0.468]
Head and Neck Squamous Cell Carcinoma vs Gastric cancer	-0.509	0.015	[-0.917, -0.101]
Other vs Gastric cancer	-0.104	0.514	[-0.416, 0.208]

**Table 4:** Multivariable meta-regression for the association of confounding factors and the hazard ratio for OS.

Variables	Coefficient	P value	95% CI
Adjustment			
Multivariate vs Univariate	-0.226	0.041	[-0.443, -0.009]
Cancer type			
Colorectal cancer vs Gastric cancer	0.050	0.687	[-0.192, 0.292]
Esophageal cancer vs Gastric cancer	-0.227	0.146	[-0.533, 0.079]
Hepatopancreatobiliary Cancer vs Gastric cancer	-0.097	0.445	[-0.346, 0.152]
Urologic cancer vs Gastric cancer	-0.327	0.344	[-1.004, 0.351]
Lung cancer vs Gastric cancer	0.095	0.496	[-0.179, 0.369]
Head and Neck Squamous Cell Carcinoma vs Gastric cancer	-0.535	0.008	[-0.928, -0.142]
Other vs Gastric cancer	-0.117	0.436	[-0.412, 0.177]

#### 3.3.2 CALLY and DFS

A total of 13 comparisons evaluated the association between the CALLY index and DFS in patients with cancer, and all included studies reported multivariable-adjusted HRs. A higher CALLY index was significantly associated with improved DFS (HR = 0.52, 95% CI: 0.46-0.58, P < 0.00001) (**Figure 2B**). This association remained consistent across subgroup analyses (**Table 5**). Although heterogeneity increased in some subgroups stratified by region, no significant heterogeneity was observed overall (I² = 0%, P = 0.52); therefore, the role of region as a source of heterogeneity could not be established. Meta-regression was not performed because no significant between-study heterogeneity was detected.

**Table 5:** Pooled HRs for DFS、PFS and RFS in subgroup analyses.

Subgroup	DFS				PFS				RFS			
	Study group	HR [95%CI]	*P* value	*I* ^2^	Study group	HR [95%CI]	*P* value	*I* ^2^	Study group	HR [95%CI]	*P* value	*I* ^2^
Total	13	0.52 [0.46, 0.58]	<0.00001	0%	11	0.39 [0.25, 0.61]	<0.0001	89%	19	0.66 [0.60, 0.72]	<0.00001	0%
Sample size												
<250	8	0.47 [0.39, 0.57]	<0.00001	0%	9	0.46 [0.31, 0.69]	0.0002	83%	8	0.55 [0.47, 0.66]	<0.00001	0%
≥250	5	0.55 [0.48, 0.64]	<0.00001	28%	2	0.20 [0.13, 0.31]	<0.00001	0%	11	0.70 [0.63, 0.78]	<0.00001	0%
Follow-up												
<30 months	NA	NA	NA	NA	6	0.39 [0.20, 0.76]	0.006	92%	1	0.53 [0.31, 0.90]	0.02	NA
≥30 months	6	0.47 [0.40, 0.55]	<0.00001	0%	2	0.21 [0.10, 0.45]	<0.0001	21%	4	0.53 [0.40, 0.70]	<0.00001	11%
Mean/median Age												
<65	5	0.47 [0.40, 0.55]	<0.00001	0%	7	0.41 [0.23, 0.72]	0.002	91%	4	0.55 [0.44, 0.69]	<0.00001	0%
≥65	8	0.58 [0.49, 0.68]	<0.00001	0%	4	0.37 [0.20, 0.69]	0.002	66%	15	0.68 [0.62, 0.75]	<0.00001	0%
Region												
China	6	0.53 [0.46, 0.60]	<0.00001	49%	3	0.44 [0.15, 1.28]	0.13	96%	3	0.59 [0.46, 0.76]	<0.0001	0%
Japan	6	0.52 [0.42, 0.65]	<0.00001	0%	2	0.21 [0.10, 0.45]	<0.0001	21%	8	0.63 [0.55, 0.73]	<0.00001	0%
Other	NA	NA	NA	NA	2	0.50 [0.34, 0.74]	0.0005	0%	1	0.43 [0.27, 0.68]	0.0004	NA
Multicenter	1	0.43 [0.23, 0.78]	0.006	NA	4	0.44 [0.26, 0.74]	0.002	54%	7	0.72 [0.64, 0.82]	<0.00001	0%
CALLY cut-off												
<1	2	0.51 [0.38, 0.68]	<0.00001	0%	4	0.44 [0.26, 0.74]	0.002	54%	1	0.93 [0.54, 1.58]	0.78	NA
1-2	NA	NA	NA	NA	4	0.48 [0.24, 0.92]	0.03	85%	4	0.65 [0.55, 0.79]	<0.00001	42%
2-3	4	0.45 [0.38, 0.54]	<0.00001	0%	2	0.43 [0.26, 0.71]	0.001	17%	3	0.59 [0.42, 0.83]	0.002	0%
3-4	2	0.48 [0.33, 0.70]	0.0001	0%	NA	NA	NA	NA	5	0.69 [0.59, 0.80]	<0.00001	0%
≥4	5	0.62 [0.51, 0.75]	<0.00001	0%	NA	NA	NA	NA	6	0.63 [0.54, 0.73]	<0.00001	0%
Treatment												
Surgery	13	0.52 [0.46, 0.58]	<0.00001	0%	4	0.24 [0.15, 0.39]	<0.00001	50%	18	0.67 [0.61, 0.73]	<0.00001	0%
Other	NA	NA	NA	NA	7	0.55 [0.37, 0.81]	0.003	78%	1	0.43 [0.27, 0.68]	0.0004	NA
Cancer type												
Gastrointestinal cancer	8	0.54 [0.47, 0.61]	<0.00001	25%	3	0.44 [0.15, 1.28]	0.13	96%	11	0.63 [0.56, 0.72]	<0.00001	10%
Hepatopancreatobiliary Cancer	2	0.48 [0.33, 0.70]	0.0001	0%	NA	NA	NA	NA	5	0.69 [0.60, 0.80]	<0.0001	0%
Urologic cancer	NA	NA	NA	NA	3	0.28 [0.16, 0.51]	<0.0001	40%	NA	NA	NA	NA
Head and Neck Squamous Cell Carcinoma	1	0.48 [0.33, 0.69]	<0.0001	NA	4	0.47 [0.28, 0.78]	0.004	51%	NA	NA	NA	NA
Other	2	0.50 [0.34, 0.73]	0.0004	17%	1	0.51 [0.30, 0.85]	0.009	NA	3	0.68 [0.53, 0.87]	0.002	0%
Tumor stage												
Nonmetastatic	NA	NA	NA	NA	2	0.21 [0.10, 0.45]	<0.0001	21%	2	0.64 [0.46, 0.89]	0.008	54%
Metastatic	1	0.60 [0.39, 0.92]	0.02	NA	7	0.53 [0.35, 0.79]	0.002	81%	NA	NA	NA	NA
Mixed	12	0.52 [0.46, 0.58]	<0.00001	0%	2	0.29 [0.11, 0.78]	0.01	85%	17	0.66 [0.60, 0.72]	<0.00001	0%

#### 3.3.3 CALLY and PFS

Progression-free survival (PFS) was reported in 11 cohorts. As shown in the forest plot (**Figure 2C**), higher pretreatment CALLY was associated with prolonged PFS compared with lower CALLY (HR, 0.39; 95% CI, 0.25-0.61; P<0.0001). Marked between-study heterogeneity was noted (I²=89%, P<0.00001); therefore, a random-effects model was applied. Subgroup analyses showed a consistent association across prespecified subgroups. However, no prognostic correlation was observed in cohorts from China (P = 0.13) or in the gastrointestinal cancer subgroup (P = 0.13), whereas significant associations were observed in the remaining subgroups (**Table 5**). Stratified analyses by sample size, follow-up duration, region, cut-off value, treatment modality, cancer type, and tumor stage showed no significant heterogeneity in several subgroups (I² < 50%), suggesting that these factors might have contributed to between-study heterogeneity.

Univariable meta-regression identified treatment modality as the main source of heterogeneity (other vs surgery: coefficient = 0.858, P = 0.003) (**Table 6**). Because of the limited number of included studies and the risk of overfitting, multivariable meta-regression was not performed. When the analysis was restricted to studies reporting multivariable-adjusted HRs, the association remained statistically significant (HR = 0.40, 95% CI: 0.25-0.64, P = 0.0001) (**Supplementary Figure S1B**).

**Table 6:** Univariate meta-regression for the association of confounding factors and the hazard ratio for PFS.

Variables	Coefficient	P value	95% CI
Sample size			
Per 1-patient increase	-0.00094	0.705	[-0.00583, 0.00394]
Follow-up			
Per 1-month increase	-0.015	0.148	[-0.035, 0.005]
Mean/Median Age			
Per 1-year increase	-0.021	0.695	[-0.126, 0.084]
CALLY cut-off			
Per 1-unit increase	-0.026	0.919	[-0.525, 0.473]
Region			
Japan vs China	-0.805	0.203	[-2.045, 0.435]
Multicenter vs China	-0.078	0.874	[-1.039, 0.883]
Other vs China	0.103	0.855	[-1.001, 1.206]
Tumor stage			
Non-metastatic vs Metastatic	-0.966	0.046	[-1.917, -0.015]
Mixed vs Metastatic	-0.627	0.107	[-1.388, 0.135]
Treatment method			
Other vs Surgery	0.858	0.003	[0.291, 1.425]
Cancer type			
Urologic cancer vs Gastrointestinal cancer	-0.521	0.347	[-1.605, 0.564]
Head and Neck Squamous Cell Carcinoma vs Gastrointestinal cancer	-0.022	0.966	[-1.024, 0.980]
Other vs Gastrointestinal cancer	0.124	0.866	[-1.322, 1.570]

#### 3.3.4 CALLY and RFS

Recurrence-free survival (RFS) was reported in 19 cohorts. As shown in the forest plot (**Figure 2D**), higher pretreatment CALLY was associated with improved RFS under a fixed-effects model (HR, 0.66; 95% CI, 0.60-0.72; P<0.00001). Subgroup analyses suggested effect modification by the CALLY cut-off value. No significant association with RFS was observed when the cut-off was <1 (P = 0.78), whereas significant associations were observed across the remaining subgroups. Between-study heterogeneity was primarily attributed to tumor stage (**Table 2**). Because no significant between-study heterogeneity was detected, meta-regression was not performed. When the analysis was restricted to studies reporting multivariable-adjusted HRs, the association remained statistically significant (HR = 0.65, 95% CI: 0.60-0.72, P < 0.00001) (**Supplementary Figure S1C**).

### 3.4 Sensitivity analysis

**Figure 3:**
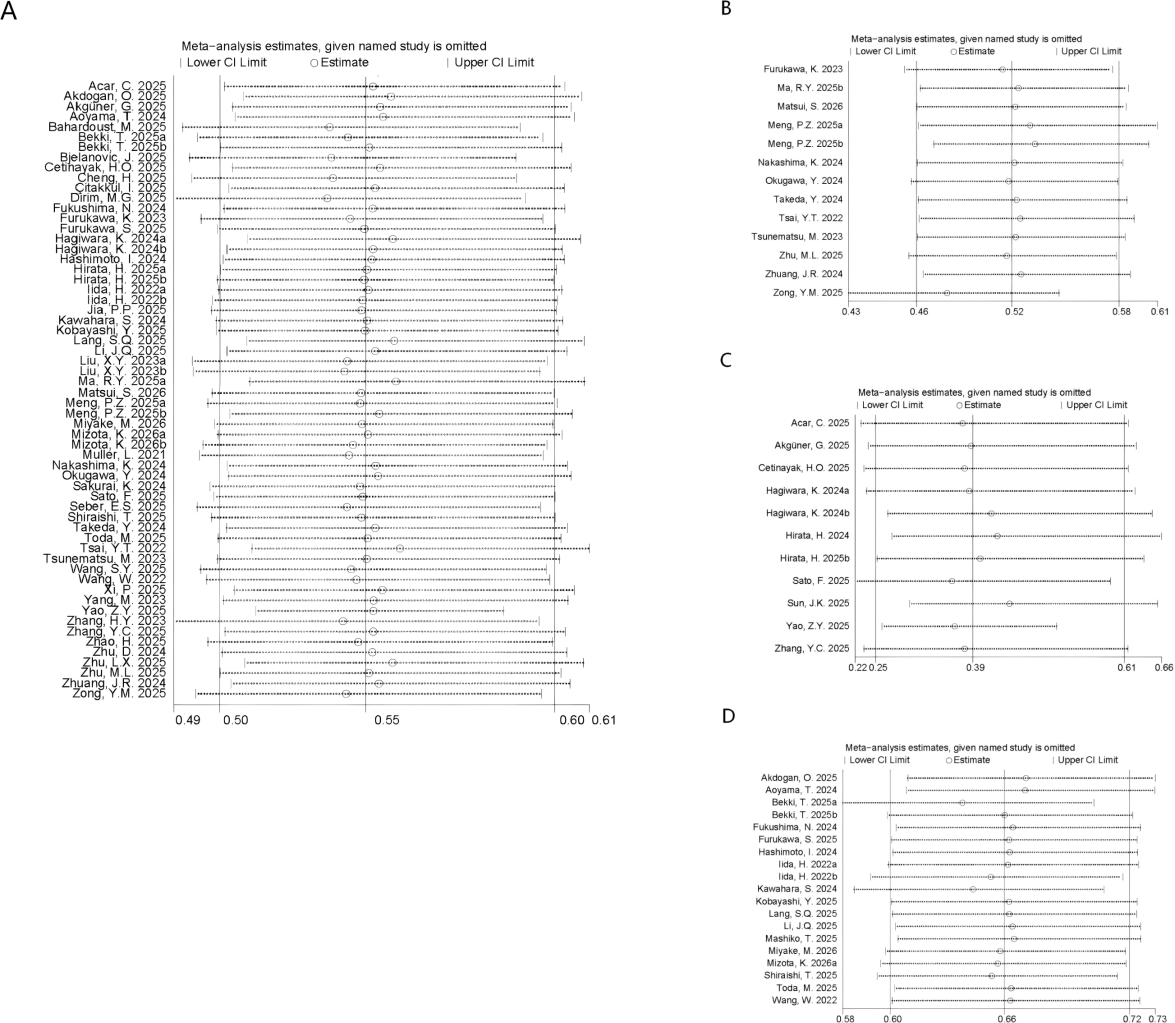
Sensitivity analysis of **(A)** OS **(B)** DFS、**(C)** PFS and **(D)** RFS.

Sensitivity analyses were implemented to appraise the reliability of the pooled estimates. After sequential removal of each publication, we identified that the pooled HRs and 95%CIs for OS (**Figure 3A**), DFS (**Figure 3B**), PFS (**Figure 3C**), and RFS (**Figure 3D**) remained significant.

### 3.5 Publication bias

**Figure 4:**
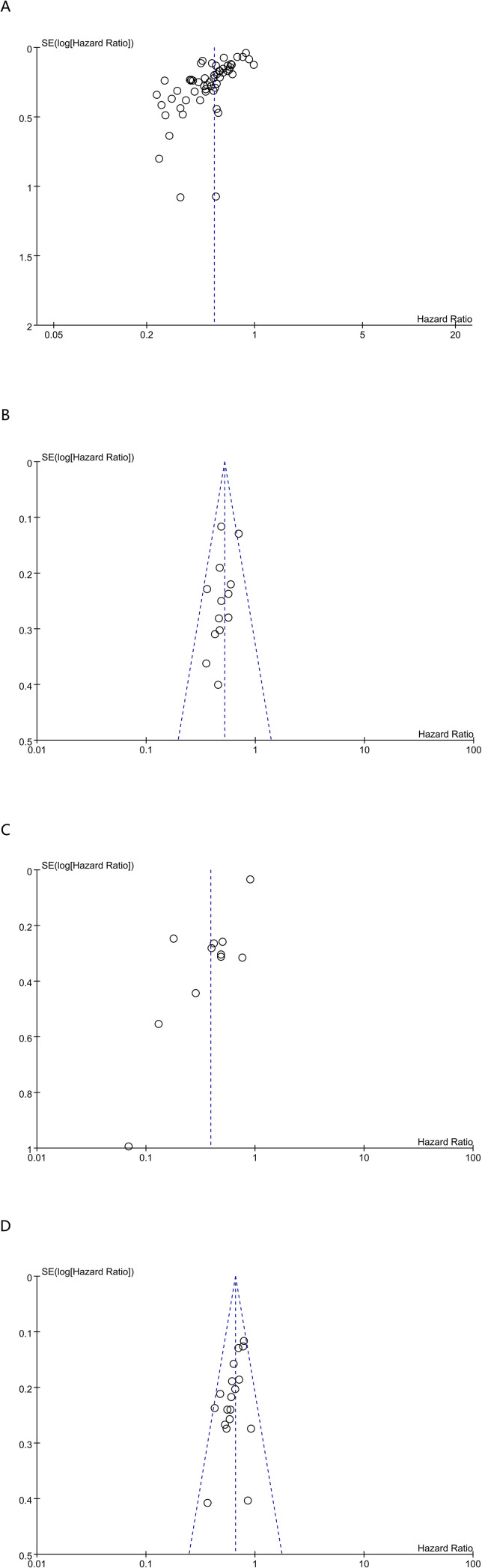
Funnel plot for the evaluation of publication bias for **(A)** OS、**(B)** DFS、**(C)** PFS and **(D)** RFS.

Publication bias for the associations of CALLY with OS, DFS, PFS, and RFS was assessed using funnel plots and Egger’s test. Egger’s test indicated no significant publication bias for DFS (P = 0.121), but suggested possible publication bias for OS (P < 0.00001), PFS (P < 0.00001), and RFS (P = 0.01). To further examine the impact of potential publication bias, a trim-and-fill analysis was performed. However, no missing studies were identified; therefore, no studies were imputed. The trim-and-fill analysis showed that the pooled estimates for OS (HR = 0.55, 95% CI: 0.50–0.60; P < 0.00001) (**Supplementary Figure S2A**), PFS (HR = 0.39, 95% CI: 0.25–0.61; P < 0.00001) (**Supplementary Figure S2B**), and RFS (HR = 0.66, 95% CI: 0.60–0.72; P < 0.00001) (**Supplementary Figure S2C**) remained largely consistent with the primary results, suggesting that potential publication bias did not materially change the overall direction of the associations. Visual inspection of the funnel plots also showed no marked asymmetry for OS (**Figure 4A**), DFS (**Figure 4B**), PFS (**Figure 4C**), or RFS (**Figure 4D**). Nevertheless, given the results of Egger’s test results, the findings for OS, PFS, and RFS were interpreted cautiously, and the potential influence of publication bias or small-study effects on the pooled estimates could not be fully excluded.

## 4 Discussion

In this meta-analysis, 64 cohorts from 56 studies comprising more than 26, 000 patients with malignancies were included to appraise the prognostic relevance of the pretreatment CALLY index for survival outcomes. Higher CALLY was linked to favorable survival endpoints in the pooled analyses. These findings support the CALLY index as a clinically accessible marker for risk categorization and prognostication in individuals with cancer.

Several differences were observed between the present meta-analysis and prior syntheses by Li et al. (8) and Zhang et al. (9). In the analysis by Zhang et al., higher CALLY was connected with significantly extended OS and DFS/RFS compared with lower CALLY. In the present study, a prognostic association with progression-free survival was also observed. Compared with earlier meta-analyses, several strengths are evident. First, our analysis included a total of 64 cohorts. In contrast, Li et al.’s meta-analysis included only 21 studies analyzing the relationship between CALLY and cancer outcomes. Incorporating additional studies would likely enhance the strength of the resulting evidence. Second, unlike previous studies, multivariable-adjusted HRs were preferentially pooled whenever feasible to better control for confounding and improve the reliability of the pooled estimates. Univariable and multivariable meta-regression analyses were also performed to explore sources of heterogeneity. Third, more granular subgroup analyses were performed, with outcomes (OS, DFS, PFS, and RFS) stratified by geographic region, sample size, CALLY cut-off values, mean age, treatment modality, tumor stage, cancer type, follow-up duration, and statistical model. This approach helps identify patient subsets in which CALLY performs best and clarifies plausible sources of heterogeneity. Finally, a broader spectrum of malignancies was represented-including colorectal, gastric, esophageal, lung, liver, and head and neck cancers, urologic cancers, pancreatic ductal adenocarcinoma, cholangiocarcinoma, ovarian and breast cancers, melanoma, and retroperitoneal sarcoma-which is essential for a more rigorous appraisal of the prognostic relevance of CALLY across cancer types.

Subgroup analyses revealed heterogeneity in the prognostic performance of CALLY across individuals with cancer and suggested several potential sources of variation. In this meta-analysis, cancer type-specific subgroup analyses showed that CALLY was not significantly correlated with prognosis in gastrointestinal cancers. This null association may have been driven by the paucity of eligible studies, which limited the reliability of cancer-specific inferences. Region-based subgroup analyses found that CALLY was not significantly linked to prognosis in publications conducted in China. The absence of a statistically significant association may reflect the scarcity of eligible studies and, more broadly, population-level differences in genetic background, dietary patterns, access to healthcare, and tumor biology. Therefore, additional international studies are needed, and CALLY measurement should be standardized to determine whether its prognostic value is consistent across regions. Our subgroup analysis by CALLY cut-off value found that the prognostic stratification ability of CALLY appeared to be more pronounced in studies using a cut-off value of ≥1. However, this finding should be interpreted cautiously, as cut-off values vary widely across studies (0.10–6.96) and are influenced by cancer type, study design, treatment context, and population characteristics. Therefore, the current evidence is insufficient to recommend a uniform clinical cut-off, and the optimal threshold requires further validation across different cancer types and clinical settings in future prospective studies.

Beyond subgroup analyses, meta-regression was performed to further investigate sources of between-study heterogeneity. The findings suggested that heterogeneity arose not only from differences in patient characteristics, but also from study design and statistical adjustment. For OS, cancer type and adjustment status were identified as independent sources of heterogeneity, indicating that the prognostic effect of CALLY was likely influenced by tumor biology, host inflammatory response patterns, and the extent of confounding control. For PFS, treatment method was identified as a significant source of heterogeneity, suggesting that the predictive value of CALLY may vary across treatment settings. Although meta-regression explained part of the between-study variation, residual heterogeneity remains, indicating that additional unmeasured or insufficiently reported factors may also contribute.

Although a higher CALLY index was associated with better survival outcomes in this study, CALLY is more appropriately interpreted as a composite prognostic indicator of the host’s overall systemic condition rather than as a mechanistic factor directly involved in tumorigenesis or tumor progression. Because the index is derived from CRP, serum albumin, and lymphocyte count, it primarily reflects systemic inflammation, nutritional reserve, and immune status, all of which are closely linked to cancer progression, treatment tolerance, and survival outcomes. Thus, the observed prognostic association is likely driven, at least in part, by the host biological state captured by these components, rather than by any direct pathogenic or protective effect of CALLY itself. Lymphocytes play a central role in antitumor immunity (65) by recognizing (66) and suppressing tumor growth and dissemination (67). Serum albumin reflects not only nutritional reserve but also, to some extent, systemic inflammatory and metabolic status (68, 69); hypoalbuminemia generally indicates limited physiologic reserve and poor overall condition (70, 71). CRP is a well-established marker of systemic inflammation (72, 73), and elevated CRP often indicates a protumor inflammatory milieu (74). Taken together, because these three components reflect immunity, nutrition, and inflammation, the clinical relevance of CALLY is best understood as an integrated measure of the overall systemic status of patients with cancer rather than as a direct surrogate for a single biological pathway.

Although important insights were provided, several limitations merit consideration. First, significant between-study heterogeneity was observed, particularly in the analyses of CALLY in relation to OS, PFS, and RFS, which may have affected the robustness and generalizability of the findings. Although subgroup analyses, meta-regression, and sensitivity analyses were performed to explore potential sources of heterogeneity, and cancer type, adjustment status, and treatment method were found to explain part of the between-study variation, residual heterogeneity remains. This suggests that additional factors, including baseline patient status, treatment details, and interstudy differences in laboratory standards, may also have contributed to the observed heterogeneity, but were not adequately reported or could not be uniformly adjusted for across studies. Second, the evidence base was largely retrospective, and susceptibility to selection and measurement bias is therefore expected. In addition, key clinical details (e.g., surgical approach and preoperative adjunctive therapies) were often incompletely reported, which constrains mechanistic interpretation and clinical translation. Third, because CALLY is a composite index derived from CRP, lymphocyte counts, and serum albumin, it is potentially influenced by intercurrent infection, comorbid conditions, medication exposure, and variability in laboratory assays; these factors were frequently not captured or adequately adjusted for in the primary studies. In addition, inconsistencies in the reporting of CALLY calculation details, measurement units, and conversion methods across some original studies may also represent a source of between-study heterogeneity. Fourth, CALLY cut-off values varied substantially across studies, ranging from 0.10 to 6.96 in the studies included in this analysis, and no universally accepted standard threshold is currently available. This wide variation reduces interstudy comparability and limits the direct generalizability and clinical applicability of CALLY across different cancer types and clinical settings. Accordingly, because different cut-off values may produce inconsistent risk stratification, the current evidence is insufficient to support the widespread use of any single fixed threshold for clinical decision-making. Fifth, although cancer type-specific subgroup analyses were performed, inference for individual tumor types remains limited because relatively few studies were available for most subgroups. Finally, although the trim-and-fill–adjusted results for OS, PFS, and RFS were largely consistent with the primary findings, Egger’s test still suggested possible publication bias or small-study effects. Therefore, the observed positive associations should be interpreted cautiously, and the possibility that unpublished negative studies may have inflated the effect estimates cannot be fully excluded.

Prospective, multicenter investigations across a large population are needed to standardize the timing of CALLY assessment, the calculation methods, and the cut-off values, as well as to systematically collect data on treatment details, complications, and concomitant medications, in order to further validate the prognostic utility of CALLY in cancer. In addition, whether interventions targeting inflammation, nutritional status, and immune function can improve the overall systemic condition of patients warrants further investigation, and the relationship between dynamic changes in CALLY and clinical outcomes should also be clarified.

## 5 Conclusion

In conclusion, this meta-analysis demonstrates that a higher pretreatment CALLY index is significantly associated with improved OS and longer DFS, PFS, and RFS in patients with cancer. These findings suggest that CALLY may be a simple and clinically useful prognostic biomarker, although further validation in high-quality prospective studies remains necessary.

## Data Availability

The original contributions presented in the study are included in the article/**Supplementary Material**. Further inquiries can be directed to the corresponding authors.
