# Supplementary figures and images for: Deciphering the potential of the C-reactive protein-albumin-lymphocyte index as a prognostic biomarker in malignancy: a systematic review and meta-analysis
Source: Front Oncol. 2026 Apr 22;16:1813296. doi: 10.3389/fonc.2026.1813296 (PMC13143774; doi:10.3389/fonc.2026.1813296)

A

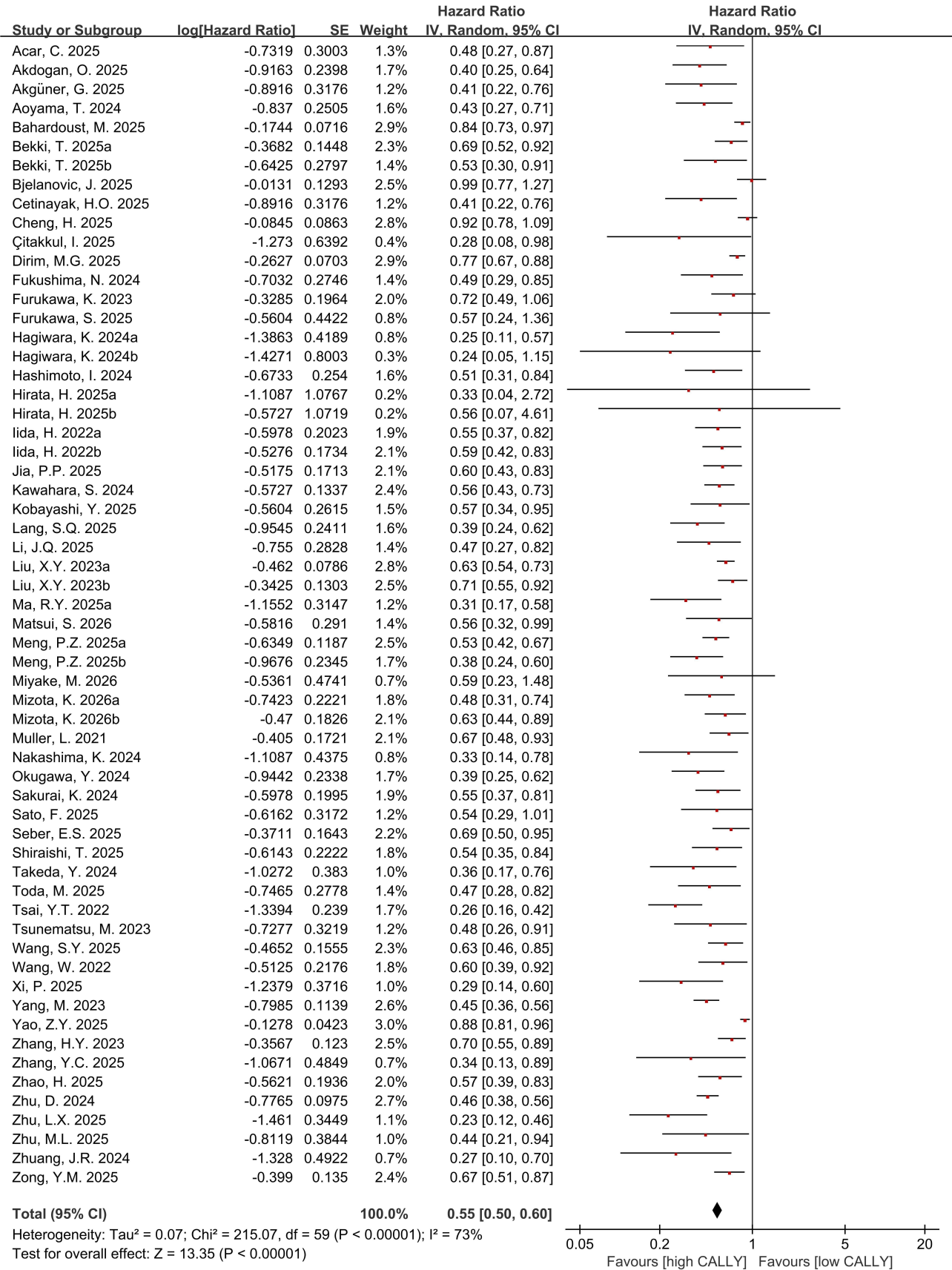

Supplement: Supplementary file 1 [file DataSheet1.pdf]

B

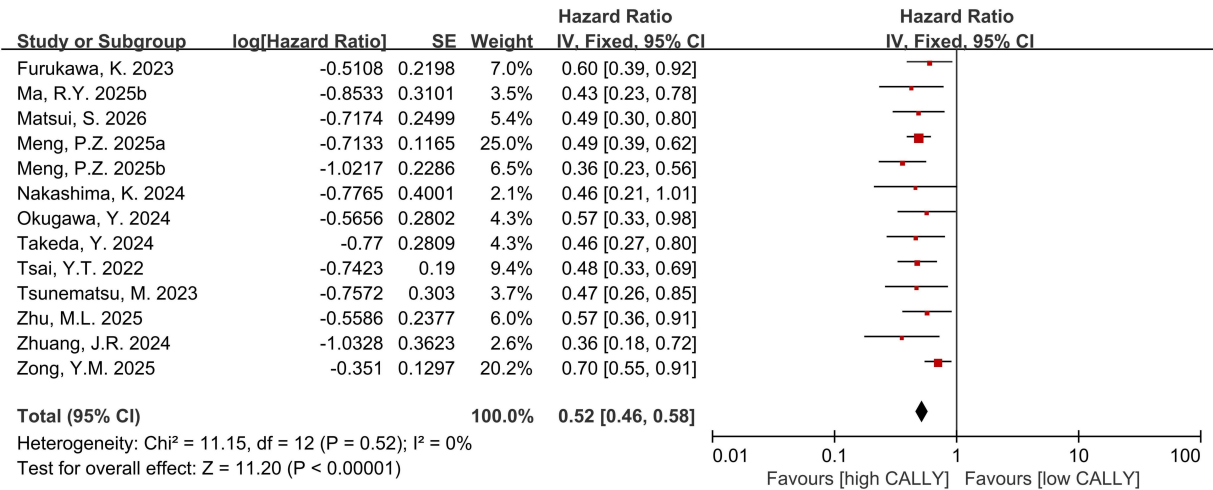

C

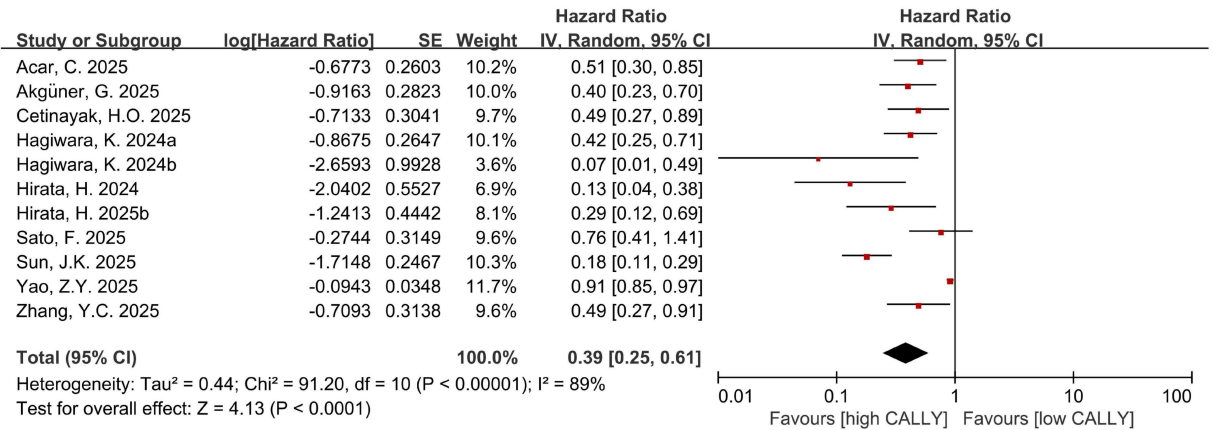

D

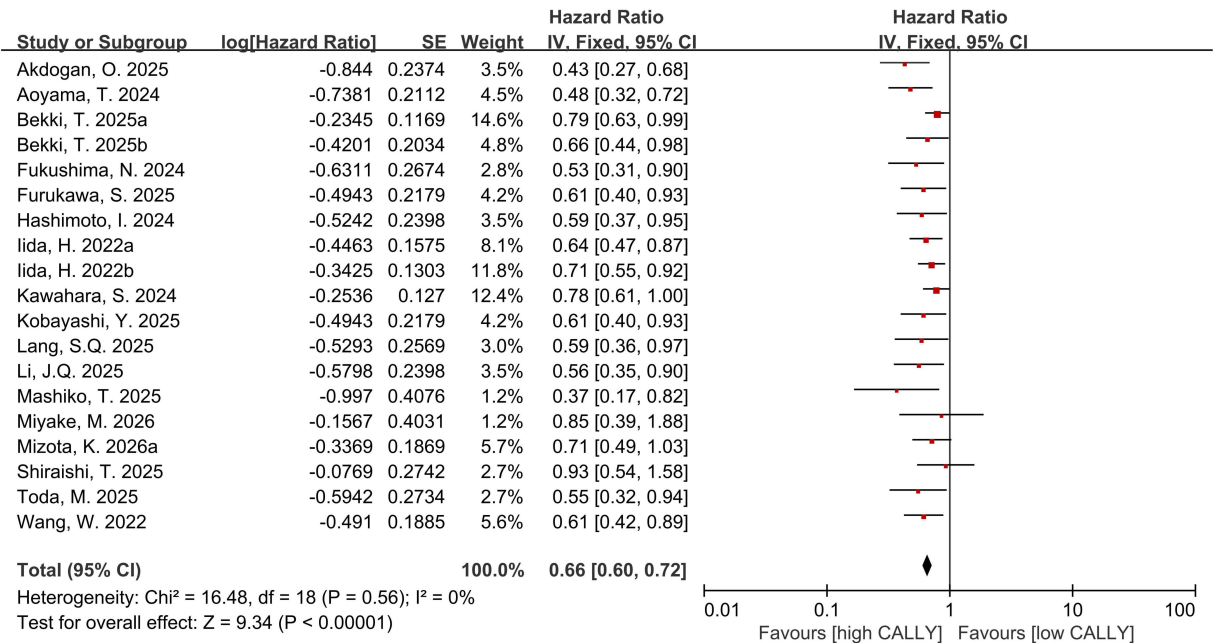

Supplement: Supplementary file 2 [file DataSheet2.pdf]

A

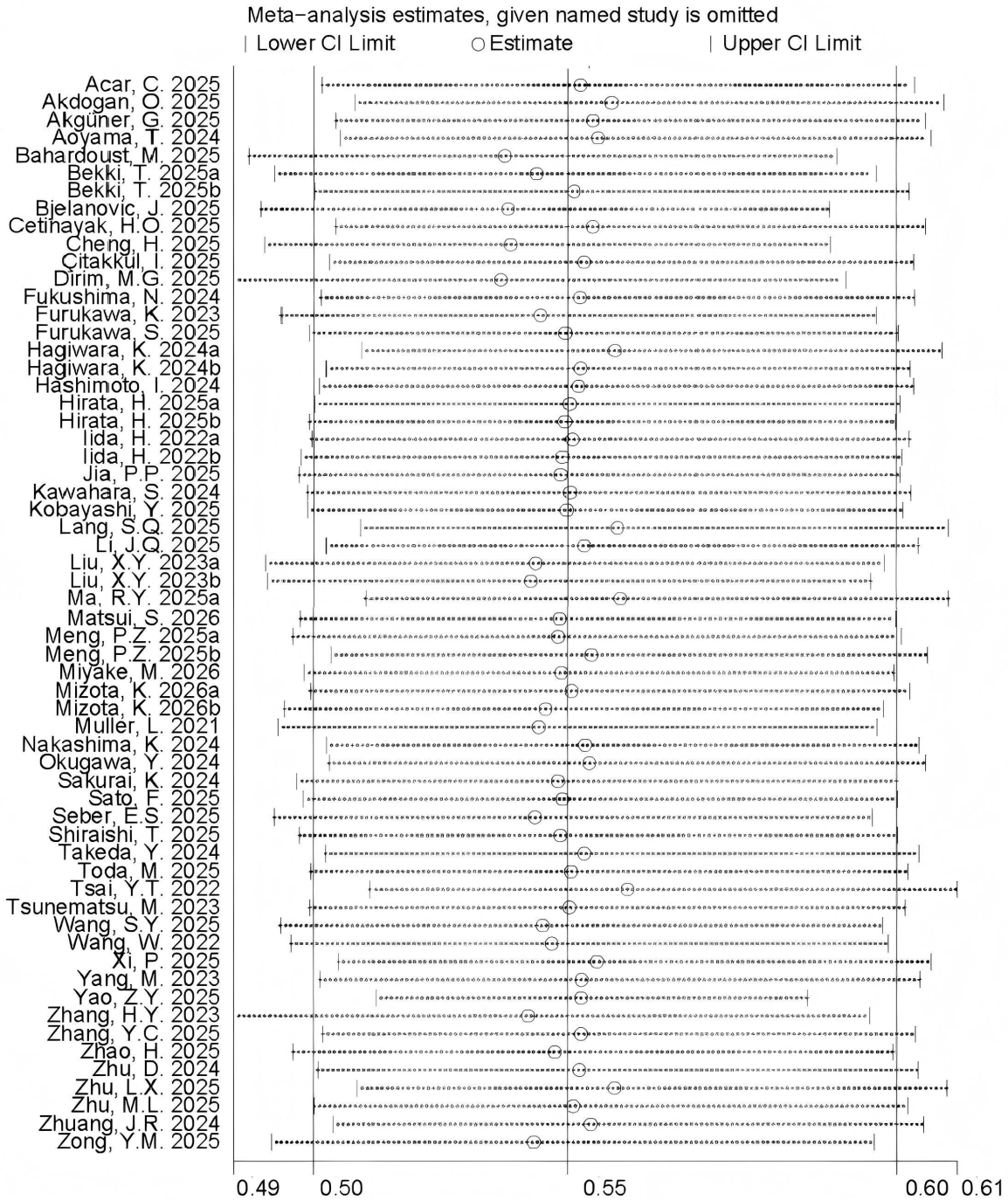

Supplement: Supplementary file 3 [file DataSheet3.pdf]

A

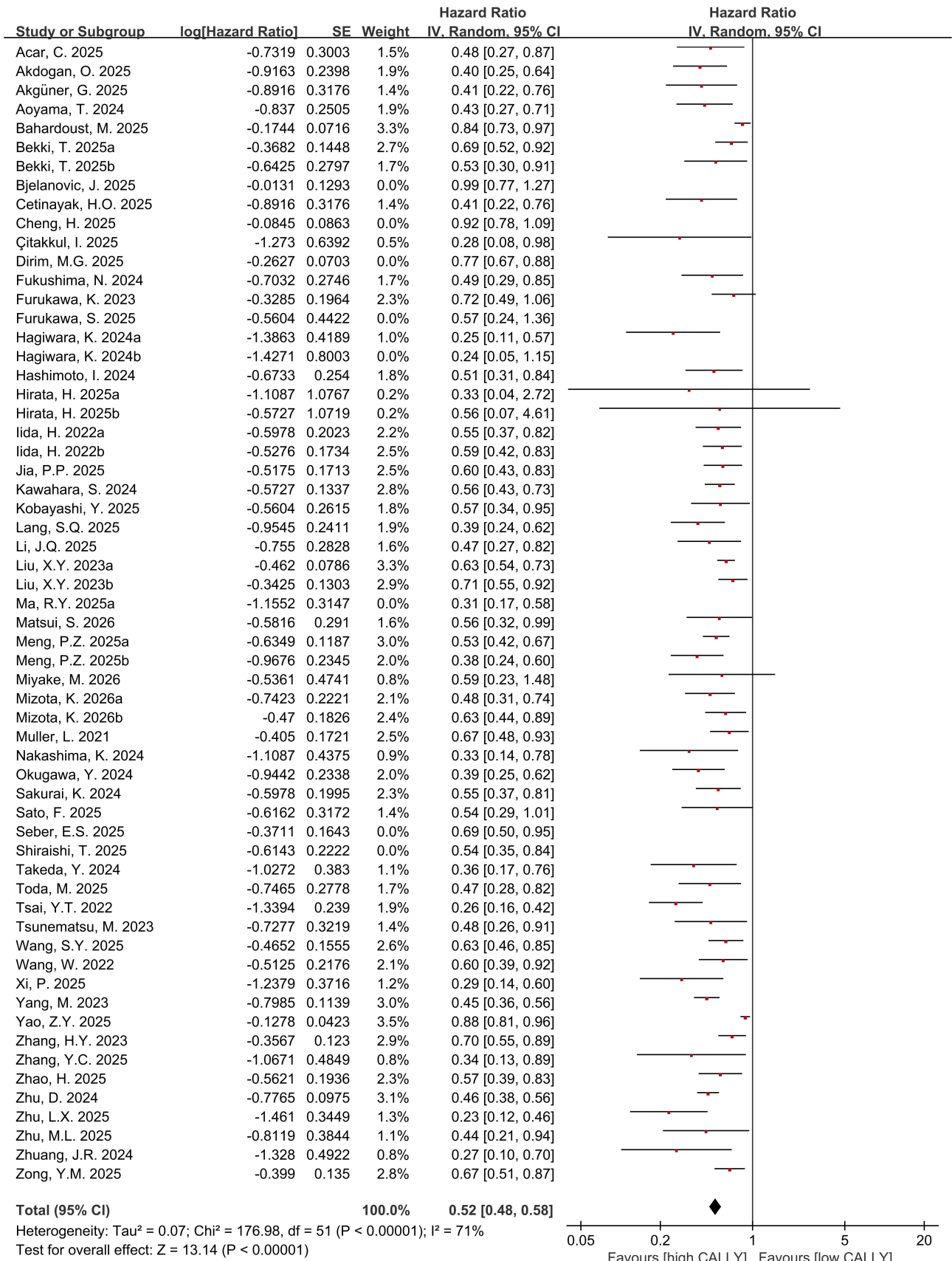

Supplement: Supplementary file 4 [file DataSheet4.pdf]

B

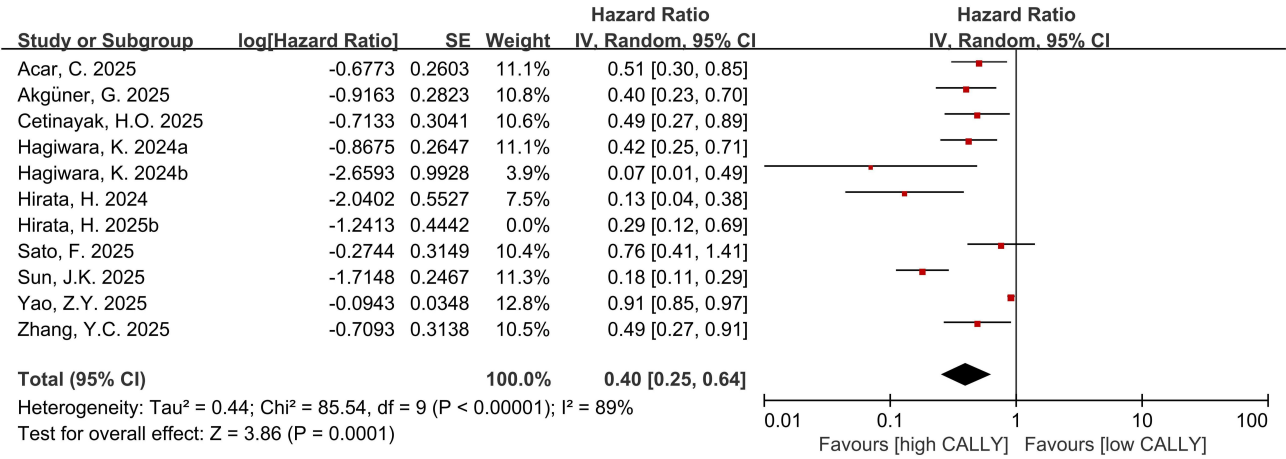

Supplement: Supplementary file 5 [file DataSheet5.pdf]

C

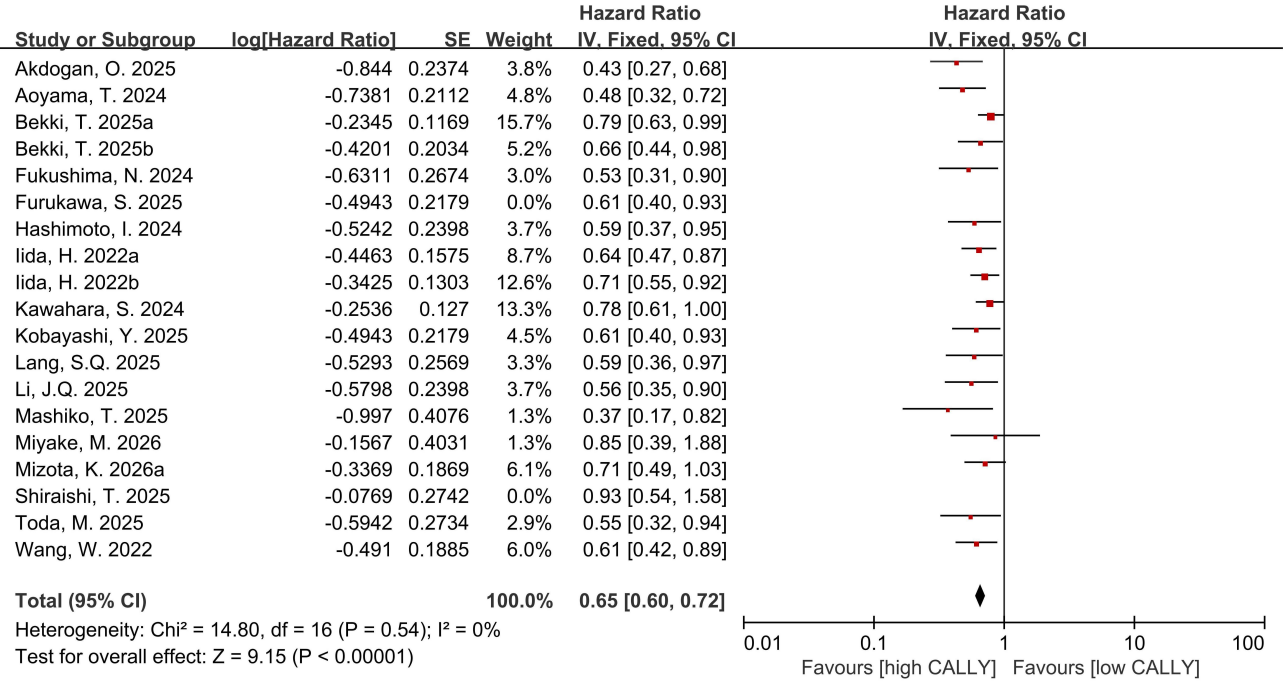

Supplement: Supplementary file 6 [file DataSheet6.pdf]

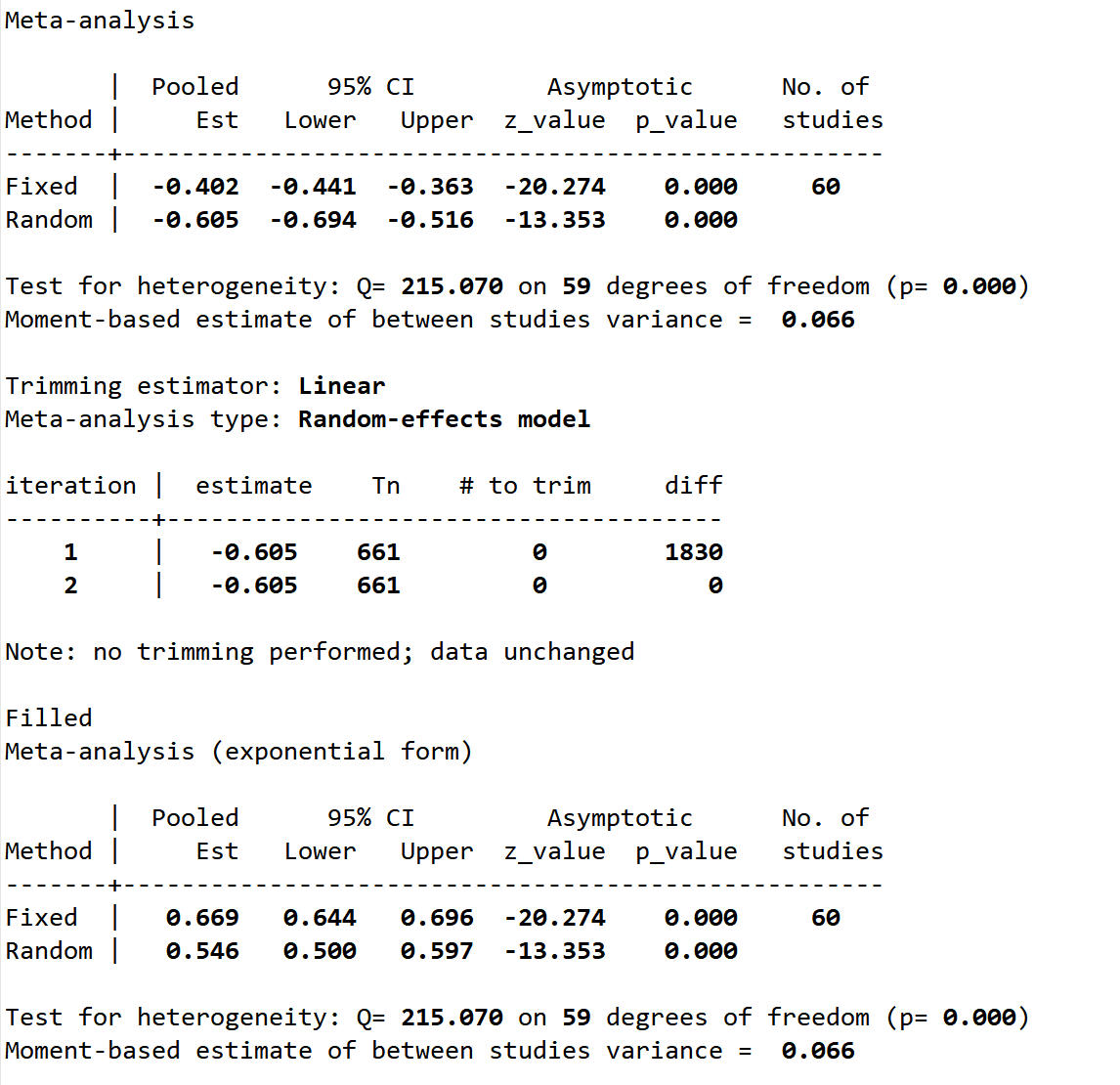

Supplement: Supplementary file 8 [file Image1.jpeg]

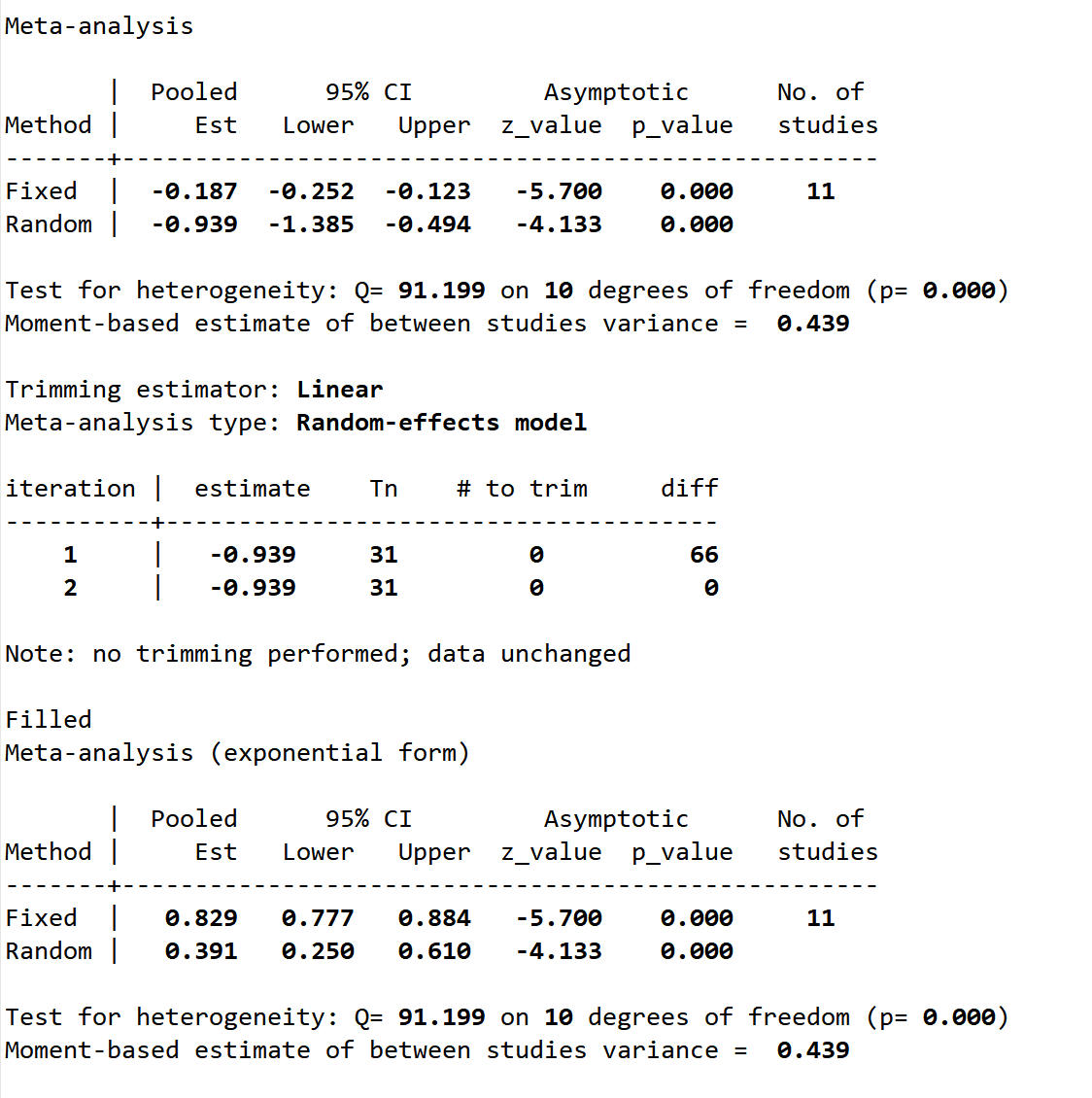

Supplement: Supplementary file 9 [file Image2.jpeg]

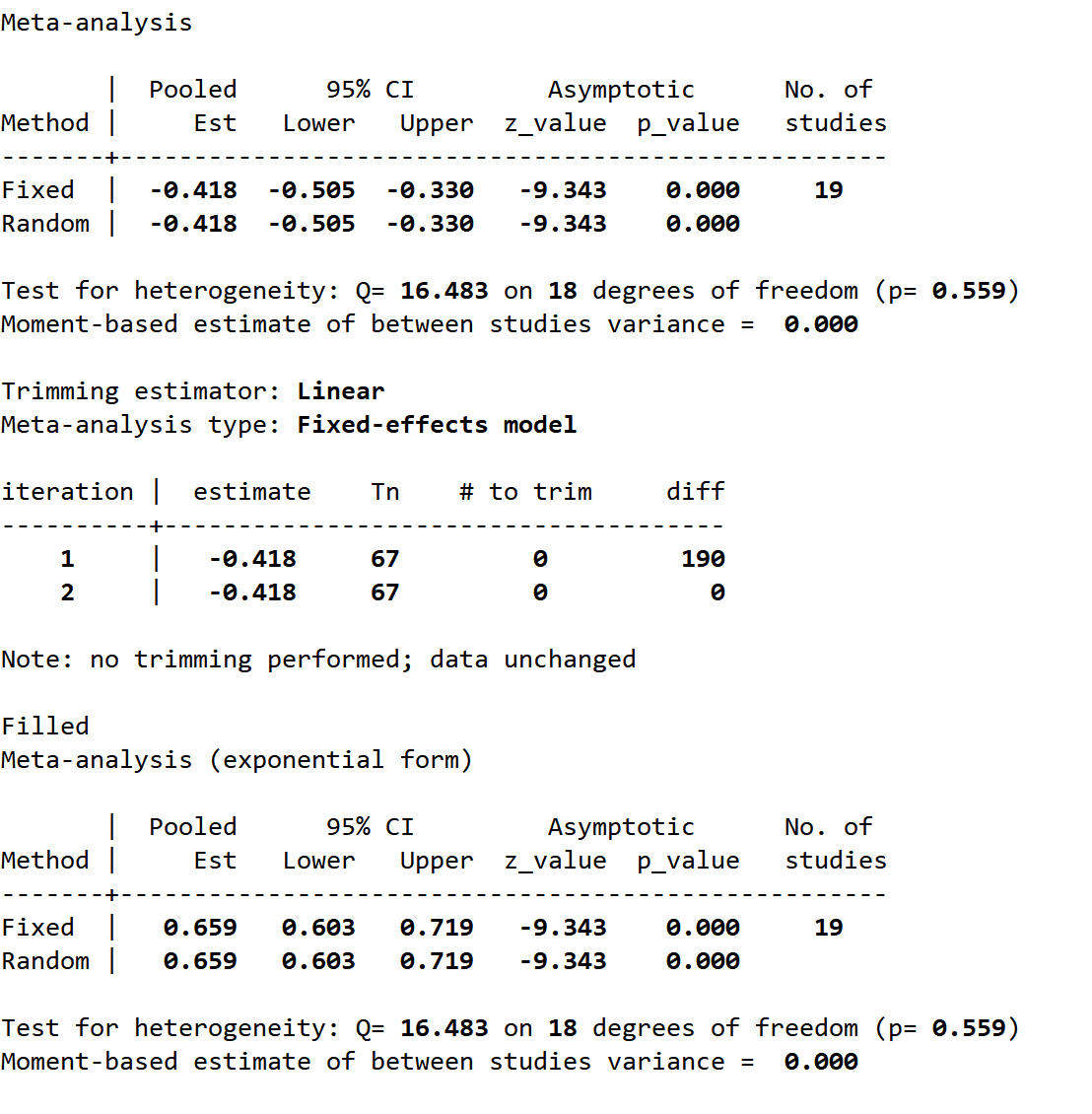

Supplement: Supplementary file 10 [file Image3.jpeg]
