# Supplementary material for: Deciphering the potential of the C-reactive protein-albumin-lymphocyte index as a prognostic biomarker in malignancy: a systematic review and meta-analysis
Source: Front Oncol. 2026 Apr 22;16:1813296. doi: 10.3389/fonc.2026.1813296 (PMC13143774; doi:10.3389/fonc.2026.1813296)
Supplement: Supplementary file 7 [file DataSheet7.docx]

**Supplementary Table S1. PRISMA 2020 checklist**

*Deciphering the potential of the C-reactive protein-albumin-lymphocyte (CALLY) index as a prognostic biomarker in malignancy: a systematic review and meta-analysis*

| **Section/topic** | **Item** | **PRISMA 2020 checklist item** | **Reported location in manuscript** | **Status / note** |
| --- | --- | --- | --- | --- |
| Title | 1 | Identify the report as a systematic review and meta-analysis. | Title | Reported |
| Abstract | 2 | See the PRISMA 2020 for Abstracts checklist. | Abstract | Reported; structured abstract present |
| Introduction | 3 | Describe the rationale for the review in the context of existing knowledge. | Introduction, paragraphs 1-4 | Reported |
| Introduction | 4 | Provide an explicit statement of the objective(s) or question(s) the review addresses. | Introduction, last paragraph | Reported |
| Methods | 5 | Specify the inclusion and exclusion criteria for the review and how studies were grouped for the syntheses. | Section 2.2 Study selection | Reported |
| Methods | 6 | Specify all databases, registers, websites, organisations, reference lists and other sources searched or consulted, and the date when each source was last searched or consulted. | Section 2.1 Search strategy | Reported |
| Methods | 7 | Present the full search strategies for all databases, registers and websites, including any filters and limits used. | Section 2.1 Search strategy; Supplementary Table S2 | Reported |
| Methods | 8 | Specify the methods used to decide whether a study met the inclusion criteria of the review, including how many reviewers screened each record and each report retrieved, whether they worked independently, and details of automation tools used in the process. | Section 2.2 Study selection | Reported |
| Methods | 9 | Specify the methods used to collect data from reports, including how many reviewers collected data from each report, whether they worked independently, any processes for obtaining or confirming data from study investigators, and details of automation tools used in the process. | Section 2.3 Data extraction | Reported |
| Methods | 10a | List and define all outcomes for which data were sought. Specify whether all results compatible with each outcome domain in each study were sought, and if not, the methods used to decide which results to collect. | Sections 2.2-2.3; survival endpoints defined in Abstract and Methods | Reported |
| Methods | 10b | List and define all other variables for which data were sought (e.g., participant and intervention characteristics, funding sources) and describe any assumptions made about missing or unclear information. | Section 2.3 Data extraction | Reported |
| Methods | 11 | Specify the methods used to assess risk of bias in the included studies, including details of the tool(s) used, how many reviewers assessed each study and whether they worked independently, and if applicable, details of automation tools used in the process. | Section 2.4 Quality assessment | Reported |
| Methods | 12 | Specify for each outcome the effect measure(s) (e.g., risk ratio, mean difference) used in the synthesis or presentation of results. | Section 2.5 Statistical analysis | Reported |
| Methods | 13a | Describe the processes used to decide which studies were eligible for each synthesis. | Sections 2.2-2.3 and 3.3 | Reported |
| Methods | 13b | Describe any methods required to prepare the data for presentation or synthesis, such as handling of missing summary statistics or data conversions. | Sections 2.3 and 2.5 | Reported |
| Methods | 13c | Describe any methods used to tabulate or visually display results of individual studies and syntheses. | Section 2.5; Figures 1-4; Tables 1-6; Supplementary Figures 1-2 | Reported |
| Methods | 13d | Describe any methods used to synthesize results and provide a rationale for the choice(s). If meta-analysis was performed, describe the model(s), method(s) to identify the presence and extent of statistical heterogeneity, and software package(s) used. | Section 2.5 Statistical analysis | Reported |
| Methods | 13e | Describe any methods used to explore possible causes of heterogeneity among study results. | Section 2.5 Statistical analysis | Reported |
| Methods | 13f | Describe any sensitivity analyses conducted to assess robustness of the synthesized results. | Section 2.5 Statistical analysis | Reported |
| Methods | 14 | Describe any methods used to assess risk of bias due to missing results in a synthesis (arising from reporting biases). | Section 2.5 Statistical analysis | Reported |
| Methods | 15 | Describe any methods used to assess certainty (or confidence) in the body of evidence for an outcome. | Not reported | Missing: no certainty assessment (e.g., GRADE) described |
| Results | 16a | Describe the results of the search and selection process, from the number of records identified in the search to the number of studies included in the review, ideally using a flow diagram. | Section 3.1; Figure 1 | Reported |
| Results | 16b | Cite studies that might appear to meet the inclusion criteria, but which were excluded, and explain why they were excluded. | Figure 1 | Reported in aggregate through full-text exclusion reasons |
| Results | 17 | Cite each included study and present its characteristics. | Section 3.1; Table 1 | Reported |
| Results | 18 | Present assessments of risk of bias for each included study. | Section 3.2; Supplementary Table S3 | Reported |
| Results | 19 | For all outcomes, present, for each study: (a) summary statistics for each group and (b) an effect estimate and its precision. | Table 1; Figure 2; Supplementary Figures 1-2 | Reported |
| Results | 20a | For each synthesis, briefly summarise the characteristics and risk of bias among contributing studies. | Sections 3.1-3.3; Table 1; Section 3.2 | Reported |
| Results | 20b | Present results of all statistical syntheses conducted. If meta-analysis was done, present for each the summary estimate and its precision and measures of statistical heterogeneity. If comparing groups, describe the direction of the effect. | Sections 3.3.1-3.3.4; Figure 2 | Reported |
| Results | 20c | Present results of all investigations of possible causes of heterogeneity among study results. | Sections 3.3.1-3.3.4; Tables 2-6; Supplementary Figure 1 | Reported |
| Results | 20d | Present results of all sensitivity analyses conducted to assess the robustness of the synthesized results. | Section 3.4; Figure 3 | Reported |
| Results | 21 | Present assessments of risk of bias due to missing results (arising from reporting biases) for each synthesis assessed. | Section 3.5; Figure 4; Supplementary Figure 2 | Reported |
| Results | 22 | Present assessments of certainty (or confidence) in the body of evidence for each outcome assessed. | Not reported | Missing: no certainty assessment reported |
| Discussion | 23a | Provide a general interpretation of the results in the context of other evidence. | Section 4 Discussion, paragraphs 1-2 | Reported |
| Discussion | 23b | Discuss any limitations of the evidence included in the review. | Section 4 Discussion, limitations paragraph | Reported |
| Discussion | 23c | Discuss any limitations of the review processes used. | Section 4 Discussion, limitations paragraph | Reported |
| Discussion | 23d | Discuss implications of the results for practice, policy, and future research. | Section 4 Discussion, final paragraph; Section 5 Conclusion | Reported |
| Other information | 24a | Provide registration information for the review, including register name and registration number, or state that the review was not registered. | Not reported | Missing: add explicit registration statement if not registered |
| Other information | 24b | Indicate where the review protocol can be accessed, or state that a protocol was not prepared. | Not reported | Missing: add protocol statement |
| Other information | 24c | Describe and explain any amendments to information provided at registration or in the protocol. | Not reported | Missing: add statement if not applicable |
| Other information | 25 | Describe sources of financial or non-financial support for the review, and the role of the funders or sponsors in the review. | Funding | Reported; funder role not explicitly described |
| Other information | 26 | Declare any competing interests of review authors. | Conflict of interest | Reported |
| Other information | 27 | Report which of the following are publicly available and where they can be found: template data collection forms; data extracted from included studies; data used for all analyses; analytic code; any other materials used in the review. | Data availability statement | Reported in general terms |
