# Supplementary material for: Deciphering the potential of the C-reactive protein-albumin-lymphocyte index as a prognostic biomarker in malignancy: a systematic review and meta-analysis
Source: Front Oncol. 2026 Apr 22;16:1813296. doi: 10.3389/fonc.2026.1813296 (PMC13143774; doi:10.3389/fonc.2026.1813296)
Supplement: Supplementary file 11 [file Table1.docx]

| Supplementary Table S1. Detailed search strategy in four databases. | |
| --- | --- |
| Database | Search strategy |
| Pubmed | (("Neoplasms"[Mesh]) OR (((((((((((((Tumors) OR (Neoplasia)) OR (Neoplasias)) OR (Neoplasm)) OR (Tumor)) OR (Cancer)) OR (Cancers)) OR (Malignant Neoplasm)) OR (Malignancy)) OR (Malignancies)) OR (Malignant Neoplasms)) OR (Benign Neoplasms)) OR (Benign Neoplasm))) AND ((((CRP-Albumin-Lymphocyte index) OR (C-reactive protein-to-albumin-to-lymphocyte index)) OR (C-reactive protein-albumin-lymphocyte index)) OR (CALLY)) |
| Embase | ((Neoplasms or (Tumors or Neoplasia or Neoplasias or Neoplasm or Tumor or Cancer or Cancers or Malignant Neoplasm or Malignancy or Malignancies or Malignant Neoplasms or Benign Neoplasms or Benign Neoplasm)) and (CRP-Albumin-Lymphocyte index or C-reactive protein-to-albumin-to-lymphocyte index or C-reactive protein-albumin-lymphocyte index or CALLY)).af. |
| Web of Science | ((Neoplasms) OR (((((((((((((Tumors) OR (Neoplasia)) OR (Neoplasias)) OR (Neoplasm)) OR (Tumor)) OR (Cancer)) OR (Cancers)) OR (Malignant Neoplasm)) OR (Malignancy)) OR (Malignancies)) OR (Malignant Neoplasms)) OR (Benign Neoplasms)) OR (Benign Neoplasm))) AND ((((CRP-Albumin-Lymphocyte index) OR (C-reactive protein-to-albumin-to-lymphocyte index)) OR (C-reactive protein-albumin-lymphocyte index)) OR (CALLY)) (Topic) |
| Chochrane | ((Neoplasms or (Tumors or Neoplasia or Neoplasias or Neoplasm or Tumor or Cancer or Cancers or Malignant Neoplasm or Malignancy or Malignancies or Malignant Neoplasms or Benign Neoplasms or Benign Neoplasm)) and (CRP-Albumin-Lymphocyte index or C-reactive protein-to-albumin-to-lymphocyte index or C-reactive protein-albumin-lymphocyte index or CALLY)).af. |
