# Supplementary material for: Deciphering the potential of the C-reactive protein-albumin-lymphocyte index as a prognostic biomarker in malignancy: a systematic review and meta-analysis
Source: Front Oncol. 2026 Apr 22;16:1813296. doi: 10.3389/fonc.2026.1813296 (PMC13143774; doi:10.3389/fonc.2026.1813296)
Supplement: Supplementary file 12 [file Table2.docx]

| Supplementary Table S2. Quality evaluation of the eligible studies with Newcastle–Ottawa scale. | | | | | | | | | |
| --- | --- | --- | --- | --- | --- | --- | --- | --- | --- |
| Study | Selection | | | | Comparability | | Outcome | | |
|  | Representative-ness | Selection of  non-exposed | Ascertainment  of exposure | Outcome not present at start | Comparability on most important factors | Comparability on other risk factors | Assessment of outcome | Long enough follow-up (median≥1 year) | Adequacy  (completeness) of follow-up |
| Liu, X.Y. et al. 2023 | * | * | * | * | - | * | * | * | * |
| Nakashima, K. et al. 2024 | * | * | * | * | - | - | * | * | * |
| Yao, Z.Y. et al. 2025 | * | * | * | * | * | * | * | * | * |
| Zhang, H.Y. et al. 2023 | * | * | * | * | * | * | * | * | * |
| Iida, H. et al. 2022 | * | - | * | * | - | - | * | * | * |
| Hagiwara, K. et al. 2024 | * | * | * | * | - | - | * | - | * |
| Çitakkul, I. et al. 2025 | * | * | * | * | - | - | * | * | * |
| Hirata, H. et al. 2025 | * | * | * | * | - | - | * | * | * |
| Wang, S.Y. et al. 2025 | - | * | * | * | - | - | * | * | * |
| Sato, F. et al. 2025 | * | * | * | * | - | - | * | * | * |
| Cetinayak, H.O. et al. 2025 | * | * | * | * | - | * | * | * | * |
| Lang, S.Q. et al. 2025 | * | * | * | * | - | - | * | * | * |
| Tsai, Y.T. et al. 2022 | * | * | * | * | - | - | * | * | * |
| Zhuang, J.R. et al. 2024 | * | * | * | * | * | - | * | * | * |
| Bahardoust, M. et al. 2025 | * | * | * | * | - | - | * | * | * |
| Akgüner, G. et al. 2025 | * | * | * | * | - | - | * | * | * |
| Acar, C. et al. 2025 | * | * | * | * | - | - | * | * | * |
| Takeda, Y. et al. 2024 | * | * | * | * | - | - | * | * | * |
| Fukushima, N. et al. 2024 | * | * | * | * | - | - | * | - | * |
| Xi, P. et al. 2025 | * | * | * | * | - | - | * | * | * |
| Sun, J.K. et al. 2025 | * | * | * | * | - | - | * | * | * |
| Kobayashi, Y. et al. 2025 | * | * | * | * | - | - | * | * | * |
| Mizota, K. et al. 2026 | * | - | * | * | - | * | * | * | * |
| Zhu, L.X. et al. 2025 | * | * | * | * | - | * | * | * | * |
| Wang, W. et al. 2022 | * | * | * | * | * | * | * | * | * |
| Meng, P. Z. et al. 2025 | * | * | * | * | - | - | * | * | * |
| Li, J.Q. et al. 2025 | * | * | * | * | - | - | * | * | * |
| Furukawa, S. et al. 2025 | - | * | * | * | - | - | * | * | * |
| Zhu, D. et al. 2024 | * | * | * | * | - | - | * | * | * |
| Mashiko, T. et al. 2025 | * | * | * | * | - | - | * | * | * |
| Miyake, M. et al. 2026 | * | * | * | * | - | * | * | * | * |
| Seber, E.S. et al. 2025 | * | * | * | * | - | - | * | - | * |
| Dirim, M.G. et al. 2025 | * | * | * | * | - | - | * | * | * |
| Toda, M. et al. 2025 | * | * | * | * | - | - | * | * | * |
| Tsunematsu, M. et al. 2023 | * | * | * | * | - | - | * | * | * |
| Furukawa, K. et al. 2023 | * | * | * | * | - | * | * | * | * |
| Muller, L. et al. 2021 | * | * | * | * | - | - | * | * | * |
| Cheng, H. et al. 2025 | * | * | * | * | * | * | * | * | * |
| Zhang, Y.C. et al. 2025 | * | * | * | * | - | - | * | * | * |
| Zhao, H. et al. 2025 | * | * | * | * | - | - | * | * | * |
| Aoyama, T. et al. 2024 | * | * | * | * | - | * | * | * | * |
| Bekki, T. et al. 2025 | * | * | * | * | - | - | * | * | * |
| Hirata, H. et al. 2024 | * | * | * | * | - | - | * | * | * |
| Shiraishi, T. et al. 2025 | * | * | * | * | - | - | * | * | * |
| Kawahara, S. et al. 2024 | * | * | * | * | - | - | * | * | * |
| Sakurai, K. et al. 2024 | * | * | * | * | - | - | * | * | * |
| Matsui, S. et al. 2026 | * | * | * | * | - | - | * | * | * |
| Zhu, M.L. et al. 2025 | * | * | * | * | - | - | * | * | * |
| Ma, R.Y. et al. 2025 | * | * | * | * | - | - | * | * | * |
| Hashimoto, I. et al. 2024 | * | * | * | * | - | - | * | * | * |
| Okugawa, Y. et al. 2024 | * | * | * | * | - | - | * | * | * |
| Zong, Y.M. et al. 2025 | * | * | * | * | - | - | * | * | * |
| Jia, P.P. et al. 2025 | * | * | * | * | - | - | * | * | * |
| Yang, M. et al. 2023 | * | * | * | * | - | - | * | * | * |
| Akdogan, O. et al. 2025 | * | * | * | * | * | * | * | * | * |
| Bjelanovic, J. et al. 2025 | * | * | * | * | - | - | * | * | * |
| *indicates criterion met; - indicates significant of criterion not met. | | | | | | | | | |
